# Construction of a *Mycobacterium smegmatis* Promoter Library for Therapeutic and Environmental Applications

**DOI:** 10.1021/acsomega.5c02222

**Published:** 2025-10-02

**Authors:** Lin Fang, Elias H. Nafziger, Min Guo, Margaret S. Saha

**Affiliations:** † Computational & Applied Mathematics & Statistics, 51433 William & Mary, Williamsburg, Virginia 23185, United States; ‡ Department of Biology, William & Mary, Williamsburg, Virginia 23185, United States; § Department of Applied Science, 8604 William & Mary, Williamsburg, Virginia 23185, United States

## Abstract

*Mycobacterium smegmatis* is a nonpathogenic species of soil-dwelling mycobacteria that shows promise as a synthetic biology chassis with both clinical and environmental applications. The development of a nonmodel chassis requires a library of regulatory genetic elements that cover a range of expression levels. Currently, most studied *M. smegmatis* promoters are characterized using single-channel reporter cassettes, which are vulnerable to extrinsic noise introduced by different culturing conditions, initial cell metabolic states, and reporter genes of choice. For constructing predictable and reliable circuits in *M. smegmatis*, this study systematically identified and analyzed 18 *M. smegmatis* promoters by using a dual-channel reporter system across different environments. Here, we show a well-characterized promoter library and a standardizable reporter plasmid construct that will allow future investigators to easily assess additional promoter elements, promoting future use of *M. smegmatis* as an effective and field-deployable chassis.

## Graphical abstract

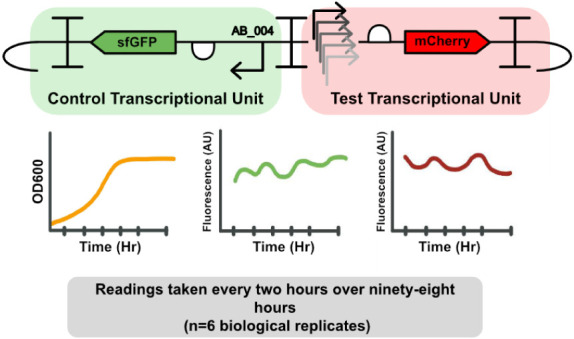

## Introduction

*Mycobacterium smegmatis* is a fast-growing, nonpathogenic species that possesses multiple features that make it a particularly attractive chassis for synthetic biology. Since the introduction of a transformable *M. smegmatis* strain, namely *M. smegmatis* MC^2^155,^1,2^ *M. smegmatis* has served as a model system for investigating the properties of pathogenic mycobacteria, including clinically important species such as *Mycobacterium tuberculosis* and nontuberculous mycobacteria pathogens such as *Mycobacterium abscessus*.^3^ With advances in recombinant DNA technology and multiomics techniques, researchers have investigated *M. smegmatis* as a vector or a host for antituberculosis and antitumor vaccines,^4–8^ as well as phage therapy.^9–12^ Additionally, due to its natural sterol metabolic pathways and its resistance to stressful bioreactor conditions, *M. smegmatis* can be effectively utilized by the biomanufacturing industry for the biotransformation of high-value pharmaceutical sterol intermediates.^13,14^ *M. smegmatis* has also been utilized as a model organism for characterizing metabolic pathways such as atmospheric carbon monoxide and hydrogen oxidation pathways.^15,16^ Additionally, due to its ubiquity across both aquatic and terrestrial biomes,^3,17,18^ *M. smegmatis* has been probed as an environmental chassis capable of operating beyond the standard laboratory setting.^19^

A collection of quantitatively characterized gene regulatory elements covering a spectrum of strengths is fundamental for the implementation of a successful synthetic biology chassis. Because of its significance, a number of mycobacterial promoters^20–23^ and promoter collections^24,25^ have been identified, constructed, and characterized using single-channel reporter cassettes. However, such single-channel assays are vulnerable to changes in global variations, such as plasmid copy numbers, different culturing conditions, and the varying state of the initial inoculated cells, undermining both the reproducibility among different laboratories and the transferability of such measurements from laboratory to field conditions.^26,27^ Multiple approaches have been proposed to enhance the reliability and reproducibility of promoter characterizations, including the utilization of a reference promoter,^26,28^ a dual-channel reporter plasmid system^27^ and multicolor fluorophore-calibrated fluorescence units.^29^ Adopting these approaches for promoter characterization, we systematically identified and analyzed 18 *M. smegmatis* promoters in different environments to create a well-characterized promoter library as well as a generalizable backbone plasmid construct that will allow future investigators to easily assess additional promoter elements and employ *M. smegmatis* as an efficacious and field-deployable synthetic biology chassis.

## Results and Discussion

### Construction of a Dual-Channel Promoter Reporter Plasmid Library

To enable reproducible measurements that could be compared across varying laboratory and environmental conditions, we designed a dual-channel fluorescence plasmid system to measure promoter activity in *M. smegmatis* (Figure 1A). This system contains two divergent transcriptional units (TU), namely, a test transcriptional unit (test TU) and a control transcriptional unit (control TU) separated by a bidirectional terminator. Both TUs are flanked by unique nucleotide sequences (UNS), which simplifies cloning procedures for future applications.^30^ The test TU contains the test promoter to be analyzed, followed by a ribosomal binding site (RBS),^31^ the mCherry coding region,^31^ and the bidirectional ttsbiB terminator.^32^ The control TU contains the reference promoter P_smyc_,^20,33^ a strong constitutive promoter followed by the same RBS sequence used for the test TU, the sfGFP coding region^34^ and the bidirectional ttsbiB terminator. The control TU enables normalization of the output of the test TU, reducing extrinsic variations introduced by factors such as different culturing conditions, metabolic burden imposed by the plasmid, or differences in assay equipment, thus allowing the accurate assessment of the intrinsic characteristics of the test promoters across varying conditions and platforms.^27^

**Figure 1:**
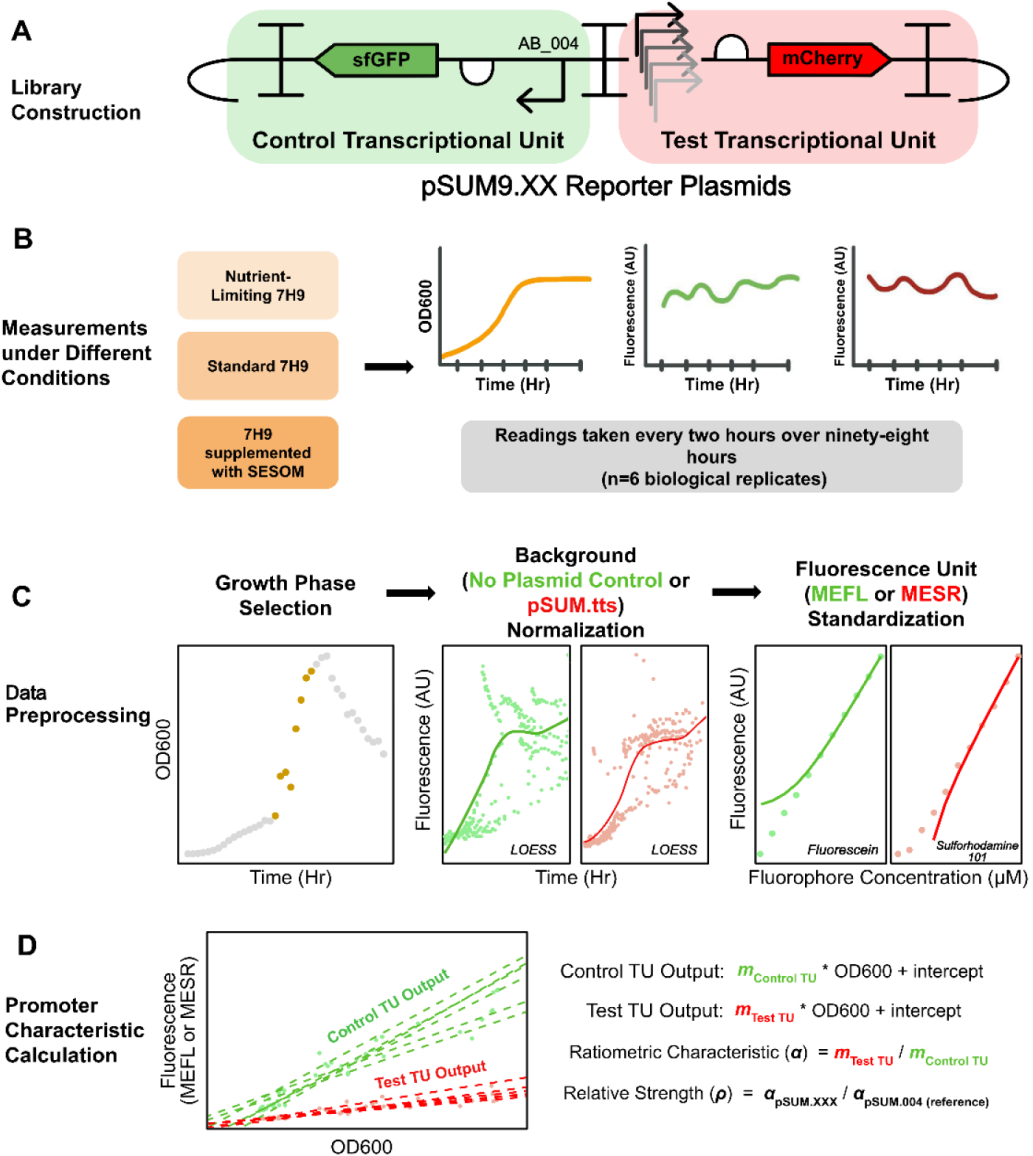
Promoter Characterization Pipeline. (A) Library Construction: pSUM9.01 - pSUM9.19 are promoter reporter plasmids constructed using 3G assembly.^35^ Each promoter reporter plasmid contains a control TU that encompasses the promoter AB_004 upstream of an sfGFP coding sequence and a test TU that encompasses a test promoter upstream of an mCherry coding sequence. The negative control plasmid pSUM.tts has a terminator in place of a test promoter. (B) Measurements under Different Conditions: *M. smegmatis* MC^2^155 transformed with promoter reporter plasmids were grown in three different conditions: standard 7H9 media, nutrient-limiting 7H9 media, and 7H9 media supplemented with soil-extracted soluble organic matter (SESOM). OD600, green fluorescence, and red fluorescence measurements were taken every two h over ninety-eight h using a Synergy H1Microplate Reader. (C) Data Preprocessing: Promoter characterization was conducted either over a 16 h exponential phase period or an early stationary phase time point. Autofluorescence of *M. smegmatis* was used to normalize control TU green fluorescence outputs. *M. smegmatis* transformed with pSUM.tts was used to normalize the test TU red fluorescence outputs. Locally Estimated Scatterplot Smoothing (LOESS) models were built to normalize the TU outputs for each experimental run. After normalization, arbitrary units (AU) of fluorescence were converted to Molecules of Equivalent Fluorescein (MEFL) or Molecules of Equivalent Sulforhodamine 101 (MESR) using standard curves. (D) The slopes of the linear models fit by the control TUs’ or the test TUs’ fluorescence outputs with respect to OD600 were denoted as the control TU outputs (*m*_control TU_) or the test TU outputs (*m*_test TU_). The ratio between *m*_test TU_ and *m*_control TU_ was denoted as the ratiometric characteristic (α) of the test promoters. The ratios between the ratiometric characteristics of the test promoters and the control construct pSUM9.04 with reference promoter AB_004-P_smyc_ were calculated to obtain relative promoter strengths (ρ). *n* = 6. For a full description of the experimental procedures, refer to the Methods section.

A total of 18 *M. smegmatis* promoters, covering a wide range of promoter strengths, were selected as test promoters for analysis in this study (Table S1). Five promoters, AB_001 (rpsL),^21^ AB_002 (ftsZ P2),^22^ AB_003 (MSMEG_5228),^23^ AB_004 (P_smyc_) and AB_006 (P_wmyc_)^20^ were selected as benchmarks from promoters previously characterized using single-channel assays in the literature. The other 13 promoters (AB_007–AB_019), not previously characterized, were identified from two published *M. smegmatis* transcriptome analyses.^36,37^ Using average reads per kilobase per million mapped reads (RPKM) values obtained with exponential phase cells (sampled after 16 h of growth) reported^36^ as a proxy for promoter strength, we selected promoters ranged from a relative RPKM (average RPKM normalized to the average RPKM of the reference promoter AB_004) of 0.004 for promoter AB_006 to 2.513 for promoter AB_012 (the MSMEG_3050 promoter) in hope for selecting a collection of promoters that cover a wide dynamic range of promoter strengths. Only transcriptional start sites (TSS) that were in agreement between both transcriptome studies were included. The 50 base pairs upstream of the TSS were then used as the test promoters.

Eighteen different reported plasmids (pSUM9.XX) were cloned and confirmed via whole plasmid sequencing (Figure 1A). OD600, green fluorescence, and red fluorescent measurements were then collected from *M. smegmatis* transformed with these constructs using a Synergy H1 Microplate Reader (Figure 1B). Data from either a 16-h exponential growth period^27^ or a single stationary phase time point^38,39^ were extracted from our time-series data for promoter strength characterization (Figure 1C). Autofluorescence of *M. smegmatis* was used to normalize the green fluorescence output of the control TU. For normalizing the red fluorescence output of the test TU, we generated a negative control plasmid (pSUM9.tts) by inserting the bidirectional terminator ttsbiB in place of a test promoter in the test TU. *M. smegmatis* transformed with pSUM9.tts does exhibit a low basal level of red fluorescence, possibly due to unintentional leakage or noise, but nonetheless higher than untransformed *M. smegmatis* (Figure S1). Thus, pSUM9.tts was included in each plate run as a negative control for the test TU background fluorescence normalization. Unique LOESS regression models,^40^ giving background green (*M. smegmatis* no-plasmid control) and red fluorescence (*M. smegmatis* with pSUM9.tts) as a function of OD600, were built for each plate run (Figure S2).^13,41^ Normalized fluorescence outputs were then converted from arbitrary units (AU) to Molecules of Equivalent Fluorescein (MEFL) or Molecules of Equivalent Sulforhodamine 101 (MESR) using standard curves (Figure 1C).^29^ For calculating the ratiometric characteristics, similar to the Rudge et al.^27^ method, the rates of change of fluorescence outputs with respect to OD600 during exponential phase were calculated and denoted as the *m*_test TU_ and *m*_contol TU_. The ratio of *m*_test TU_ and *m*_contol TU_ was denoted as the unitless ratiometric characteristic α. The ratio of the α_test promoter_ and α_reference promoter (AB_004‑Psmyc)_ was then denoted as the relative promoter strength of the corresponding test promoter (Figure 1D).

### Dual-Channel Characterization Allows Capture of Intrinsic Promoter Properties

Overall, even with stringent normalization, there is a presence of noise and variation across our data set, indicated by the varying timing of transitions between growth phases and overall ranging growth patterns across constructs (Figure 2A). Additionally, control TU outputs (m_contol TU_) differ across different promoter reporter plasmids (Figure 2B), showing the necessity of the internal normalization using a dual-channel characterization system. Computing the ratiometric characteristics reduces the variation across biological replicates (Figure 2C), dissipating outlier data points’ influences, and significantly reduces the coefficient of variation (CV) of promoter measurements compared to single-channel outputs (Figure 2D, Table S2 and S3). These results indicate that our promoter characterization pipeline reduces extrinsic variations rooted in both technical limitations^42,43^ and the inherent stochasticity in gene expression,^44^ allowing us to capture the intrinsic properties of test promoters.

**Figure 2:**
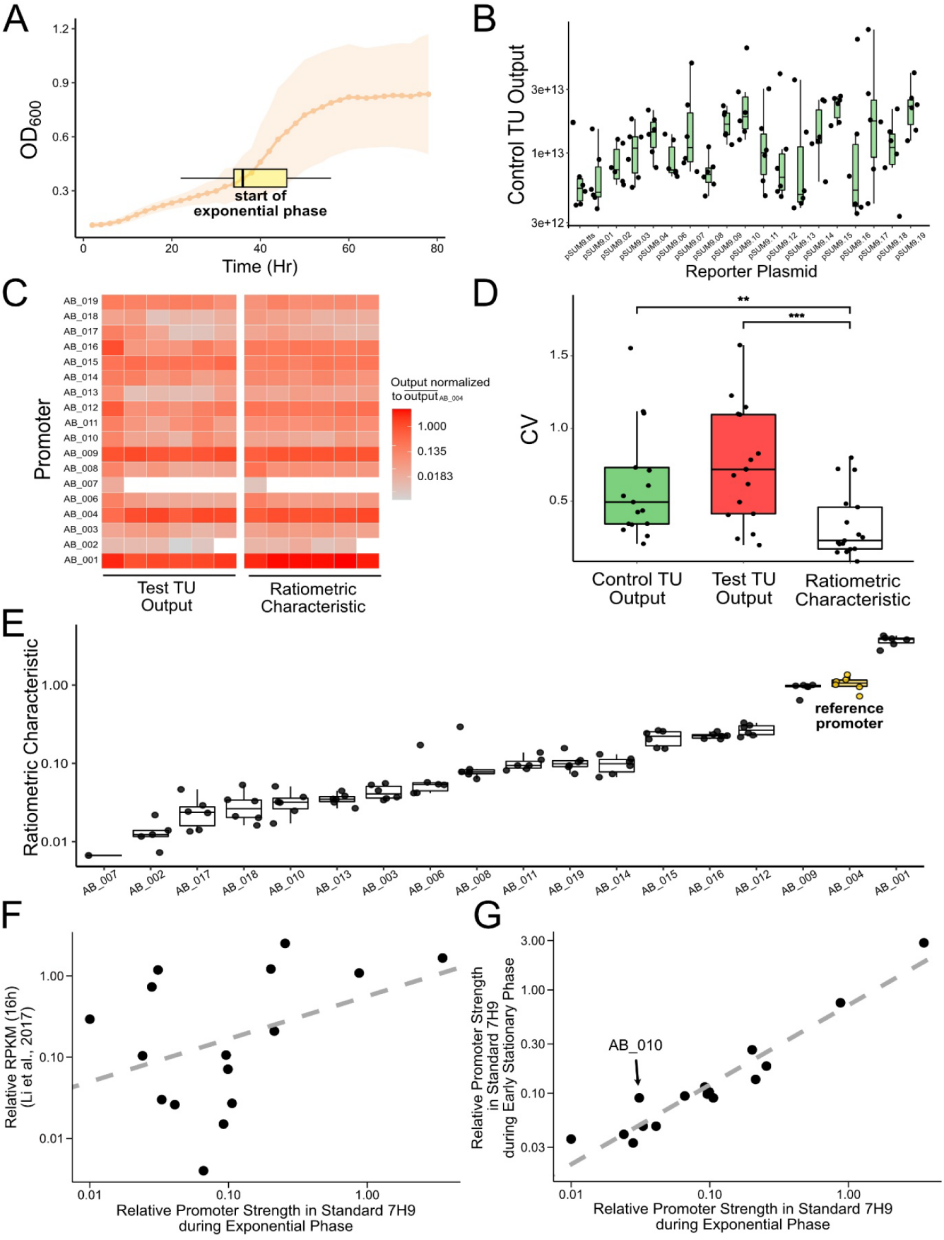
Characterization of Test Promoters in Standard 7H9Media. (A) Average growth curve of *M. smegmatis* transformed with reporter plasmids. *n* = 108, ribbon = ± SD. The horizontal box plot displays the distribution of the initial time points of the exponential growth phase. *n* = 108. (B) Controls the TU outputs of all test promoter constructs. Green fluorescence data points were normalized using untransformed *M. smegmatis*. Normalized control TU outputs exhibit differences across test promoter constructs (Kruskal–Wallis rank-sum test, *p* < 0.001). *n* = 6. (C) Heat map of test TU outputs and ratiometric characteristics across test promoters (*y* axis) with six biological replicates (*x* axis). Both metrics were normalized by the mean of AB_004’s corresponding metric. Six data points of negative values were omitted after the log transformation of the color scale (five data points of AB_007 and one data point of AB_002). (**D)** Comparison of the coefficient of variations (CV) of promoter characteristic measurements using single-channel control TU outputs, single-channel test TU outputs, and dual-channel ratiometric characteristics. CV is defined as SD/mean. Each point represents a different promoter construct. The distribution of ratiometric characteristics displays a CV lower than both single-channel outputs across constructs. The Kruskal–Wallis rank-sum test, **p = 0.0054 for control TU output vs ratiometric characteristic, ***p < 0.001 for test TU output vs ratiometric characteristic. *n* = 6 per promoter construct. (E) Ratiometric characteristics of all test promoters. Reference promoter AB_004-P_smyc_ is highlighted in yellow. Six data points of negative values were omitted after the log transformation of *y* axis (five data points of AB_007 and one data point of AB_002). Each point represents one replicate. *n* = 6. (F) Relative promoter strength obtained in this study did not correlate with relative RPKM reported in the literature.^36^ Dashed lines represent the linear relationship between the relative promoter strength and relative RPKM on a log–log scale. AB_007 is omitted due to its nonpositive average ratiometric characteristic. Adjusted *R* ^2^ = 0.0945. (G) Test promoters’ relative promoter strengths obtained from the exponential growth phase and the early stationary phase generally align with each other. Dashed lines represent the linear relationship between the relative promoter strengths obtained from the two growth phases on a log–log scale. AB_007 is omitted due to its nonpositive average ratiometric characteristic. Adjusted *R* ^2^ = 0.9166.

Eighteen promoters were characterized with ratiometric characteristics (p) ranging approximately 2.5 orders of magnitude (Figure 2E). The characterization of the five previously characterized promoters (AB_001–AB_004, AB_006) from the literature generally aligns, in relative terms, with the assessment of promoter activity in this study. AB_001 was reported to be a strong promoter^21^ and was characterized by this study to possess the highest promoter strength among all promoters tested. Using single-channel TUs, AB_004 was characterized to be driving a 16-fold stronger expression of GFPuv compared to the promoter AB_006.^20^ This relationship was also observed in this study. The average promoter activity calculated for AB_004 is 15.04 times higher than that of AB_006 (Figure 2E and Table S3). AB_003 was characterized by Uhía et al.^23^ to be a TetR family regulator-repressible promoter located upstream of the gene encoding a 3-*b*-hydroxysteroid dehydrogenase involved in the cholesterol catabolic pathway. In this study, the relative strength of AB_003 was measured to be 0.041, a relatively weak promoter, potentially due to the absence of cholesterol in the lab culture conditions of *M. smegmatis* used in this study.

While there is general agreement between the promoter strengths of the previously characterized promoters described above and our assessment of promoter strength, it is notable that the promoter strengths quantified in our study did not typically correlate with the relative RPKM values reported in the literature^36^ (Figures 2F and S3). For example, based on the relative RPKM, five promoters were reported to be stronger than our reference promoter AB_004. However, we found that only one of these five promoters (AB_001) displayed a relative promoter strength greater than 1. For example, AB_012 had a relative RPKM of 2.513, the second highest among test promoters; however, in our study, promoter AB_012 had a relative promoter strength of 0.256. Similarly, AB_010 (the MSEMG_1919 promoter) was reported to be a strong promoter according to its relative RPKM (1.184) but was characterized as a relatively weak promoter in our study.

On the other hand, AB_014 (the MSMEG_3976 promoter) was reported to be a weak promoter (relative RPKM of 0.015), while AB_018 (the MSMEG_5180 promoter) was reported to be a medium-strong promoter (relative RPKM of 0.732). However, our study indicates exactly the reverse, with AB_014 exhibiting significantly higher expression levels than AB_018. These discrepancies suggest that the RPKM values from RNA-seq experiments are not suitable for predicting the strength of the corresponding promoter but are rather only appropriate for generating preliminary hypotheses to be tested. This could be due to various post-transcriptional modifications affecting TUs’ outputs, which RPKM does not take into account,^45^ exclusion or truncation of upstream regulatory elements during promoter sequence extraction,^46^ or the different culturing conditions the cells encounter across different experimental procedures and laboratories. Such discrepancies were also noted during other promoter characterization studies with *M. smegmatis*,^36^ *Methylotuvimicrobium buryatense*,^47^ *Rhodobacter sphaeroides*,^48^ *Staphylococcus aureus*,^46^ and *Streptomyces albus*,^49^ highlighting the necessity of stringent wet lab experimental validations similar to this study.

Notably, AB_007 displayed a negative average ratiometric characteristic of −0.018 due to pSUM9.07’s test TU mCherry expression being indistinguishable from the background red fluorescence of the negative control plasmid pSUM.tts (Table S3). This correlates to AB_007’s relative RPKM^36^ of 0.005, the second lowest among our test promoters. This suggests one of the shortcomings of fluorescence protein-based assays—having a high detection limit unsuitable for characterizing weak promoters.^50^ Downstream analysis of AB_007 was still carried out due to the non-negative ratiometric characteristics displayed by some biological replicates.

Recognizing the merits of promoters that are stable across growth phases for both biomanufacturing purposes^39^ and field-deployable applications,^51^ we utilized our data set to characterize the test promoters’ transcriptional activity in the early stationary phase. Using the method similar to Yu et al.,^39^ we calculated the ratiometric characteristic of test promoters over a single time point during the stationary phase (Table S4). We chose to not use the Rudge et al.^27^ method here because of its assumption of a linear relationship between the cells’ fluorescent protein production rate and growth rate no longer holds as cells transition to the stationary phase. Test promoters’ ratiometric characteristics obtained from the exponential and stationary phases are generally consistent (Figure 2G). Notably, AB_010 shifted from being the 13th strongest promoter during the exponential phase to being the ninth during the stationary phase (Figure S4). AB_010, the MSMEG_1919 promoter, is located upstream of the MSMEG_1919 gene encoding a WhiB-like transcriptional factor.^36^ WhiB-like family proteins are intrinsically disordered transcriptional factors widely distributed in the Actinobacteria phylum and actinobacteriophages, and typically act as regulators in response to oxidative and nitrosative stresses.^52^ Currently, there is no evidence in literature to support WhiB-like transcriptional factors’ expression is upregulated as *M. smegmatis* transits to the stationary phase, but there is data showing that whcB, a *Corynebacterium glutamicum* whiB homologue, had a 3-fold expression in the stationary phase over the exponential phase.^53^

### Promoter Characterization in Differential Culturing Conditions

To assess both the reliability of the dual-channel system and the variability of the test promoters’ activity across different culturing conditions, we then characterized this collection of promoters with two additional growth media that mimic the scarcity of essential nutrients found in soil environments (7H9, 0.02% glycerol, w/o albumin–dextrose (AD) supplement (NL-7H9)) and the presence of metabolites distinct from commonly used lab compounds such as glucose and yeast extract (7H9, 0.2% glycerol, supplemented with AD supplement and soil-extracted soluble organic matter (SESOM) (Soil-7H9))^54,55^ (Figures 1B and 3A). Notably, *M. smegmatis* transformed with test constructs displays different growth profiles across the three culturing conditions. In general, *M. smegmatis* grown in NL-7H9 halts exponential growth at lower OD600 values compared to others (Figure 3B). Normalized relative strengths of each test promoter, combining measurements from all growth conditions, displayed lower coefficients of variation (CVs) compared to the outputs of single-channel test TUs, indicating that the ratiometric promoter characteristics are less affected by the extrinsic variations introduced by differential growth conditions than single-channel fluorescence outputs (Figure S5). We observed that across growth conditions fold changes of ratiometric characteristics of test promoters measured in NL-7H9 or in Soil-7H9 with respect to their corresponding ratiometric characteristics measured in 7H9 are moderately conserved (Figure 3C), suggesting that promoter measurements were heavily influenced by universal factors dependent on culturing conditions. Additionally, we observed that test promoters generally exhibit higher levels of noise under nutrient-limiting conditions, which matches Sureka et al.^56^’s results that, in *M. smegmatis*, an increase in noise in basal gene expression contributes to cells’ transition to alternative transcriptional landscapes in response to stress. After normalizing the ratiometric characteristics of test promoters using the mean ratiometric characteristic of the reference promoter AB_004, the majority of the test promoters displayed a consistent relative strength across different culturing conditions (Table S5), suggesting the necessity of normalizing against a reference promoter, which aids in screening out media-dependent global factors.^57^

**Figure 3:**
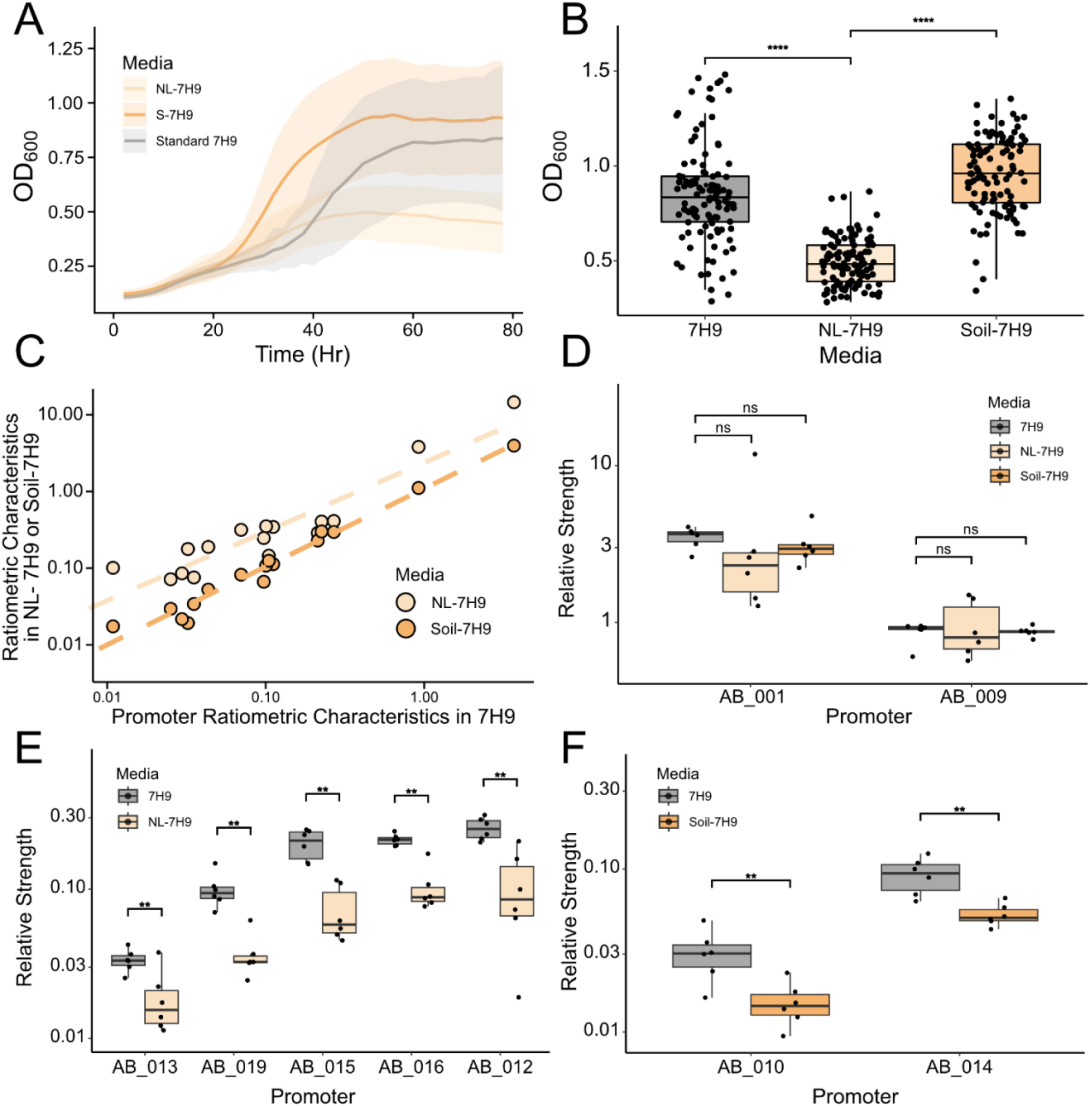
Characterization of Test Promoters in Nutrient-Limiting 7H9 Medium and 7H9 Supplemented with SESOM. (A) Average growth curves of *M. smegmatis* transformed with reporter plasmids in 7H9, NL-7H9, and Soil-7H9. *n* = 108 per growth condition, ribbon = ± SD. (B) Average OD600 when *M. smegmatis* culture exits the exponential growth phase in three different culturing conditions. *M. smegmatis* cultures in NL-7H9 enter the stationary phase at a lower OD600 than cultures in other culturing conditions. Wilcoxon rank-sum test, ****p < 0.0001 for 7H9 vs NL-7H9 and 7H9 vs Soil-7H9. (C) Fold-change of ratiometric characteristics of test promoters in NL-7H9 or Soil-7H9 relative to their ratiometric characteristics obtained from standard 7H9 cultures is, in general, conserved. Dashed lines represent linear relationships between average ratiometric characteristics of test promoters in NL-7H9 or Soil-7H9 and ratiometric characteristics in 7H9 on a log–log scale. AB_007 is omitted due to its nonpositive average ratiometric characteristic. Adjusted R^2^ = 0.8576 for NL-7H9 vs 7H9, adjusted *R* ^2^ = 0.9698 for Soil-7H9 vs 7H9. **(D**) Two strong promoters, AB_001 and AB_009, have statistically nonsignificant relative promoter strengths across three culturing conditions. Wilcoxon rank-sum test, *p* > 0.05 for all comparisons. *n* = 6. (E) Five promoters, AB_012, AB_013, AB_015, AB_016, and AB_019, exhibit differential relative promoter strengths between 7H9 cultures and NL-7H9 cultures. *n* = 6. Wilcoxon rank-sum test, **p < 0.0043 for AB_012, *p < 0.041 for AB_013, **p < 0.0022 for AB_015, **p < 0.0022 for AB_016, **p < 0.0022 for AB_019. (F) Two promoters, AB_010 and AB_014, exhibit differential relative promoter strengths between 7H9 cultures and Soil-7H9 cultures. *n* = 6. Wilcoxon rank-sum test, **p < 0.0087 for AB_010, **p < 0.0043 for AB_014.

Overall, our characterization pipeline, which includes normalization steps using an internal control TU and a reference promoter construct, enables us to capture the intrinsic characteristics of the test promoters across the culturing conditions. Two strong promoters, AB_001 and AB_009, displayed consistent promoter dynamics across all growth conditions (Figure 3D), making them ideal candidate parts for constructing synthetic systems intended to be deployed into soil systems. Both AB_001 and AB_009 are located upstream of genes encoding ribosomal proteins (RPs),^36^ suggesting that RP-expressing promoters could be a reservoir for discovering strong constitutive promoters that maintain consistent expression levels when experiencing different conditions.^49^ Multiple sequence alignment of the six strongest promoters characterized in this study revealed a clear −10 box matching the mycobacterial sigma factor SigA motif from previous studies,^58,59^ providing empirical insights for the rational design of additional strong mycobacterial promoters (Figure S6). Comparing constructs grown in 7H9 and NL-7H9, 5 promoters (AB_012, AB_013, AB_015 (the MSMEG_4272 promoter), AB_016 (the MSMEG_4276 promoter), and AB_019 (the MSMEG_6182 promoter)) displayed a lower activity that was statistically significant (Figures 3E and S7). Similarly, 2 out of 18 promoters (AB_010, AB_014 (the MSMEG_3976 promoter)) displayed significantly lower promoter activity when comparing cultures grown in standard 7H9 and Soil-7H9 (Figures 3F and S7). The majority of promoters that exhibit differential activity possess low-to-medium strength under standard laboratory conditions and could potentially be regulated promoters that are further repressed under nutrient starvation or the presence of antimicrobial metabolites in soil.^60,61^ These differentially expressed promoters could be useful for monitoring bacterial stress levels and controlling gene expression in response to stress level fluctuations.

## Conclusion

In this study, we constructed a modular system for synthetic biologists to use for accurate *M. smegmatis* promoter characterization under varying conditions (Figures S8 and S9, Sequences S1 and S2). We constructed 18 promoter-reporter plasmids (Sequences S3–S20) and provided an assessment of 18 *M. smegmatis* promoters under laboratory and soil-mimicking environments, where we identified promoters suitable for an array of different circuit-design applications. This study lays the foundation for both the characterization and development of *M. smegmatis* as a fieldable synthetic biology chassis and the construction of field-deployable *M. smegmatis* systems.

## Methods

### Bacterial Strains and Growth Conditions

Both *Escherichia coli* strain 5α (NEB) and DH5α (Thermo Fisher) were used as chassis for plasmid assembly. For growing *E. coli* liquid culture, Luria–Bertani (LB) medium was used. Cells were placed in incubation at 37 °C with shaking at 250 rpm for 14–16 h until saturation. For plating, LB plates with a 1.5% agar were used. When necessary, antibiotics were added to the medium at the following final concentrations: kanamycin, 50 μg/mL; chloramphenicol, 25 μg/mL. *Mycobacterium smegmatis* MC^2^155 (ATCC no. 700084) was used for promoter characterization. For starting liquid cultures, *M. smegmatis* was grown at 37 °C and 250 rpm in Middlebrook 7H9 broth supplemented with 0.2% glycerol, 10% albumin–dextrose supplement, and 3–4 beads per tube to prevent cell clumping. Cultures were grown for 36 h to an optical density measured at 600 nm (OD600) of approximately 0.8–1.0 and diluted 100-fold into either standard 7H9 broth (0.2% glycerol, 10% albumin–dextrose supplement), nutrient-limiting 7H9 broth (0.02% glycerol, w/o albumin–dextrose supplement), or 7H9 supplemented with soil-extracted soluble organic matter (SESOM). SESOM was prepared as described by Valian et al.^55^ For preparing 2X SESOM, 200 g of air-dried soil was suspended in 500 mL of 20 mM MOPS buffer with shaking at 200 rpm at 37 °C for 1 h. Liquid extract was then filtered through coffee filter paper and a 0.22 μM vacuum filtration system (Thermo Scientific). 2X SESOM was then mixed with 2X 7H9 broth with a 1:1 ratio to obtain the 1X 7H9 supplemented with SESOM. For plating, 7H9 plates with 1.5% agar were used. For all *M. smegmatis* cultures, antibiotics were added to the medium at the following final concentrations: carbenicillin, 50 μg/mL; cycloheximide, 10 μg/mL. When necessary, kanamycin (35 μg/mL) was supplemented into the medium for plasmid-transformant selection.

### Physical Parts Isolation

Fragments comprising individual parts were either isolated from *M. smegmatis* MC^2^155 genomic DNA or available plasmids (Addgene) using PCR, or ordered as linear double-stranded gene blocks (gBlock, Integrated DNA Technologies) (Tables S6 and S7). All promoter fragments are flanked with BsaI cut sites to facilitate downstream seamless insertion into the backbone destination vectors using the Golden Gate assembly. Individual parts were inserted into modified pSB1C3 ^62^ vectors and stored in *E. coli* DH5α. All parts were confirmed by Sanger sequencing.

### Plasmid Assembly

The overall strategy for plasmid assembly for this study is the 3G assembly described by Halleran et al.^35^ Individual parts were assembled into transcriptional units via the Golden Gate assembly. The constructed transcriptional units were then inserted into a linearized pSUM-lacZ-gfp backbone^63^ via Gibson assembly. At each step, the assembly reaction products were transformed into *E. coli* DH5α. Transformation products were plated on LB agar plates with the corresponding antibiotics for transformant selection. Colony PCR was used to confirm the successful assembly. Confirmed colonies were then inoculated into the LB liquid medium. All constructs were then extracted and sequence confirmed (Plasmidsaurus, Louisville, KY).

### Electroporation of *Mycobacterium smegmatis*

*M. smegmatis* electrocompetent cells were prepared and transformed similar to protocols described by Beggs et al.^64^ Briefly, single *M. smegmatis* MC^2^155 colonies were picked, grown to an OD600 of 0.8–1.0, washed with 10% glycerol and harvested with centrifugation at 5000 rpm 4 times. 1 μg (maximum volume of 5 μL) of DNA was transformed into electrocompetent *M. smegmatis* using an eporator system (Eppendorf). For each electroporation, one pulse was delivered (time constant: 5.0 ms; field strength: 12 kV/cm). Cells were allowed to recover in supplemented 7H9 broth for 1 h and plated on 7H9 plates with kanamycin (35 μg/mL).

### Microplate Reader Fluorescence Measurements

For each promoter construct, six *M. smegmatis* transformant colonies were picked and grown in supplemented 7H9 media with kanamycin (35 μg/mL) until an OD600 of 0.8–1.2 was reached. 50 μL of the culture were then diluted 100-fold into 5 mL of supplemented 7H9 media with kanamycin (35 μg/mL). For each measurement, 200 μL of each promoter reporter plasmid culture was loaded onto a 96-well plate. Blank media, untransformed *M. smegmatis*, *M. smegmatis* transformed with the negative control plasmid pSUM.tts, and a dilution series of fluorophore calibrants were also included for each plate for calibration and data normalization. A Synergy H1 Microplate Reader was used to measure OD600, green fluorescence (excitation wavelength: 488 nm; emission wavelength: 530 nm; bandwidth: 20 nm; gain: 65), and red fluorescence (excitation wavelength: 570 nm; emission wavelength: 620 nm; bandwidth: 20 nm; gain: 100). Readings were taken every two h over ninety-eight h.

### Readout Data Normalization and Analysis

Arbitrary fluorescence units were converted into molecules of equivalent fluorophore calibrants (Molecules Equivalent of Fluorescein (MEFL) or Molecules Equivalent of Sulforhodamine-10 (MESR)).^29^ For each plate run, two 2-fold serial dilutions of fluorescein (0.01 to 10.00 μM) and sulforhodamine-10 (0.001 to 2 μM) were included, and standard curves specific to each plate run were constructed. The green autofluorescence of untransformed *M. smegmatis* MC^2^155 was used to normalize the outputs of the control TUs, and the red fluorescence of *M. smegmatis* transformed with the negative control construct pSUM9.tts was used to normalize the outputs of the test TUs. Locally estimated scatterplot smoothing (LOESS) regression models^40,41,65^ were built to predict the two negative control strains’ TUs’ fluorescence outputs at different OD600 values. LOESS models were constructed with the loess function in RStudio.^66^ Six biological replicates of both negative control strains were included for each plate run for constructing LOESS models specific to each plate. OD600 values were normalized by using the median OD600 of the appropriate media specific to each plate. The overall normalization process is as follows:

1 $$\frac{F_{\mathrm{sample}}\left(t\right)-F_{\mathrm{LOESS}}\left(OD_{\mathrm{sample}}\left(t\right)\right)}{OD_{\mathrm{sample}}\left(t\right)-OD_{\mathrm{blank}}}$$

where

● *F*_sample_ *(t)* = Fluorescence output at time point *t*

● *F*_LOESS_ *(OD*_sample_ *(t))* = Negative control’s fluorescence output predicted by the corresponding LOESS regression model

● *OD*_sample_ *(t)* = OD600 at time point *t*

● *OD*_blank_ = OD600 of the corresponding media

The method from Rudge et al.^27^ was used to calculate the promoter characteristics. For both TUs, the slope *m* of the fluorescence (normalized with LOESS model outputs) vs OD600 curves during the exponential growth phase was calculated. The exponential growth phase was defined as the eight consecutive time points that had the maximum rate of range in OD600 with respect to time. Linear models were fit using OD600 and fluorescence outputs. The slopes of linear models fitted using control TUs’ green fluorescence outputs with respect to OD600 were denoted as *m*_controlTU_. The slopes of linear models fitted using test TUs’ red fluorescence outputs with respect to OD600 were also denoted as *m*_testTU_. The ratio between m_testTU_ and *m*_controlTU_ was denoted as the ratiometric characteristic (α) of a test promoter:

2 $$\alpha =\frac{m_{test\;TU}}{m_{control\;TU}}$$

And to calculate the relative promoter strength (ρ):

3 $$\rho =\frac{\alpha_{\mathrm{test promoter}}}{\alpha_{\mathrm{reference promoter}\left(AB\_004-P\mathrm{smyc}\right)}}$$

For determining promoter strength at the stationary phase, similar to the approach described by Yu et al.,^39^ the ratio between the normalized outputs of the two TUs at a single time point (6 h after the end of the 16-h exponential growth period) was calculated and normalized to the OD600 value of that time point. The early stationary phase promoter strength of AB_004 was used as the reference to obtain the relative promoter strengths of test promoters at the early stationary phase.

### Data Analysis

The fluorescence levels were analyzed using either the Wilcoxon rank-sum test or the Kruskal–Wallis rank-sum test. Significance was set at *p* value <0.05. Data analyses were performed using R Statistical Software (v4.3.3; R Core Team 2024) and RStudio^66^ with packages including ggbump,^67^ ggplot2,^68^ ggpubr,^69^ and tidyverse.^70^ Multiple sequence alignment was performed using Kalign^71^ and visualized using Jalview.^72^

## Supplementary Material





## Notes

Background Test TU fluorescence, LOESS models for TU normalization, change of rank order of promoter measurements in different culturing conditions and different growth phases, CV of promoter measurements, promoter sequence alignment, promoter strengths across culturing conditions, plasmid sequences and maps used by this study, reference list for both Supporting Information files (PDF)
Promoter information, sequence, single-channel TU outputs and dual-channel ratiometric characteristics, comparison of promoters across culturing conditions, parts, primers, and plasmids used by this study (XLSX)

## #

Systems, Synthetic, and Physical Biology, Rice University, 6100 Main St., Houston, TX 77005, United States

## ∇

Virology, Immunology, and Microbiology, Boston University Chobanian and Avedisian School of Medicine, 620 Albany St., Boston, MA 02218, United States.

## ○

Biostatistics and Health Data Science, University of Minnesota Twin Cities Graduate School, 321 Johnston Hall 101 Pleasant Street SE, Minneapolis, MN 55455, United States.

## Abbreviations

Control TU: control transcriptional unit
LOESS: locally estimated scatterplot smoothing regression
OD600: optical density measured at 600 nm
MEFL: molecules equivalent of fluorescein
MESR: molecules equivalent of sulforhodamine-10
RBS: ribosomal binding site
RPKM: reads per kilobase per million mapped reads
SESOM: soil-extracted solubilized organic and inorganic matter
Test TU: test transcriptional unit

## References

[1] Snapper S. B., Lugosi L., Jekkel A., Melton R. E., Kieser T., Bloom B. R., Jacobs W. R. (1988). Lysogeny and
transformation in mycobacteria: Stable expression of foreign genes. Proc. Natl. Acad. Sci. U. S. A.

[2] Snapper S. B., Melton R. E., Mustafa S., Kieser T., Jacobs W. R. (1990). Isolation and characterization of efficient plasmid
transformation mutants of *Mycobacterium smegmatis*. Mol. Microbiol.

[3] Xie W., Wang L., Luo D., Soni V., Rosenn E. H., Wang Z. (2023). *Mycobacterium
smegmatis*, a Promising Vaccine Vector
for Preventing TB and Other Diseases: Vaccinomics Insights and Applications. Vaccines.

[4] Sweeney K. A., Dao D. N., Goldberg M. F., Hsu T., Venkataswamy M. M., Henao-Tamayo M., Ordway D., Sellers R. S., Jain P., Chen B. (2011). A recombinant *Mycobacterium smegmatis* induces potent bactericidal immunity
against Mycobacterium tuberculosis. Nat. Med.

[5] Guo X.-Q., Wei Y.-M., Yu B. (2012). Recombinant *Mycobacterium
smegmatis* expressing Hsp65–hIL–2 fusion protein
and its influence on lymphocyte function in mice. Asian Pacific J. Tropical Med.

[6] de
los Angeles García M., Borrero R., Marrón R., Lanio M. E., Canet L., Otero O., Kadir R., Suraiya S., Zayas C., López Y. (2013). Evaluation of specific humoral immune response and cross reactivity
against Mycobacterium tuberculosis antigens induced in mice immunized
with liposomes composed of total lipids extracted from *Mycobacterium
smegmatis*. BMC Immunol.

[7] Kadir N. A., Sarmiento M. E., Acosta A., Norazmi M. N. (2016). Cellular and humoral
immunogenicity of recombinant *Mycobacterium smegmatis* expressing Ag85B epitopes in mice. Int. J.
Mycobacteriol.

[8] Jian W., Li X., Kang J., Lei Y., Bai Y., Xue Y. (2018). Antitumor
effect of recombinant *Mycobacterium smegmatis* expressing
MAGEA3 and SSX2 fusion proteins. Exp Ther Med.

[9] Broxmeyer L., Sosnowska D., Miltner E., Chacón O., Wagner D., McGarvey J., Barletta R. G., Bermudez L. E. (2002). Killing
of Mycobacterium avium and Mycobacterium tuberculosis by a Mycobacteriophage
Delivered by a Nonvirulent Mycobacterium: A Model for Phage Therapy
of Intracellular Bacterial Pathogens. J. Infect.
Dis.

[10] Danelishvili L., Young L. S., Bermudez L. E. (2006). In Vivo
Efficacy of Phage Therapy
for Mycobacterium avium Infection As Delivered by a Nonvirulent Mycobacterium. Microb. Drug Resist..

[11] Azimi T., Mosadegh M., Nasiri M. J., Sabour S., Karimaei S., Nasser A. (2019). Phage therapy as a
renewed therapeutic approach to
mycobacterial infections: A comprehensive review. Infect. Drug. Resist.

[12] Dedrick R. M., Smith B. E., Cristinziano M., Freeman K. G., Jacobs-Sera D., Belessis Y., Whitney Brown A., Cohen K. A., Davidson R. M., van Duin D. (2023). Phage Therapy of Mycobacterium Infections:
Compassionate Use of Phages in 20 Patients With Drug-Resistant Mycobacterial
Disease. Clin. Infect. Dis.

[13] Fernandez-Cabezon L., Galan B., Garcia J. L. (2017). Engineering *Mycobacterium
smegmatis* for testosterone production. Microb. Biotechnol.

[14] Galan B., Uhia I., Garcia-Fernandez E., Martinez I., Bahillo E., de la Fuente J. L., Barredo J. L., Fernandez-Cabezon L., Garcia J. L. (2017). *Mycobacterium smegmatis* is a suitable
cell factory for the production of steroidic synthons. Microb. Biotechnol.

[15] Grinter R., Kropp A., Venugopal H., Senger M., Badley J., Cabotaje P. R., Jia R., Duan Z., Huang P., Stripp S. T. (2023). Structural
basis for bacterial energy extraction
from atmospheric hydrogen. Nature.

[16] Kropp A., Gillett D. L., Venugopal H., Gonzalvez M. A., Lingford J. P., Jain S., Barlow C. K., Zhang J., Greening C., Grinter R. (2025). Quinone extraction drives atmospheric
carbon monoxide oxidation in bacteria. Nat.
Chem. Biol.

[17] Smeulders M. J., Keer J., Speight R. A., Williams H. D. (1999). Adaptation of *Mycobacterium smegmatis* to stationary phase. J. Bacteriol.

[18] Joseph
Antony Sundarsingh T., Ranjitha J., Rajan A., Shankar V. (2020). Features of
the biochemistry of *Mycobacterium smegmatis*, as a
possible model for Mycobacterium tuberculosis. J. Infect. Public Health.

[19] Fleeharty M. S., Carline K. B. R., Tchadi B. V., Shockey B. B., Holley E. C., Saha M. S. (2025). Survival and spread of engineered *Mycobacterium
smegmatis* and associated mycobacteriophage in soil microcosms. Appl. Environ. Microbiol.

[20] Kaps I., Ehrt S., Seeber S., Schnappinger D., Martin C., Riley L. W., Niederweis M. (2001). Energy transfer
between fluorescent proteins using a co-expression system in *Mycobacterium smegmatis*. Gene.

[21] Kenney T. J., Churchward G. (1996). Genetic analysis of the *Mycobacterium
smegmatis* rpsL promoter. J. Bacteriol.

[22] Roy S., Anand D., Vijay S., Gupta P., Ajitkumar P. (2011). The ftsZ Gene
of *Mycobacterium smegmatis* is expressed Through Multiple
Transcripts. Open Microbiol J.

[23] Uhia I., Galan B., Medrano F. J., Garcia J. L. (2011). Characterization
of the KstR-dependent promoter of the gene for the first step of the
cholesterol degradative pathway in *Mycobacterium smegmatis*. Microbiology.

[24] Spratt J. M., Britton W. J., Triccas J. A. (2003). Identification of
strong promoter
elements of *Mycobacterium smegmatis* and their utility
for foreign gene expression in mycobacteria. FEMS Microbiol. Lett.

[25] Sun H., Yang J., Song H. (2020). Engineering
mycobacteria artificial
promoters and ribosomal binding sites for enhanced sterol production. Biochem. Eng. J.

[26] Kelly J. R., Rubin A. J., Davis J. H., Ajo-Franklin C. M., Cumbers J., Czar M. J., de Mora K., Glieberman A. L., Monie D. D., Endy D. (2009). Measuring the activity of BioBrick
promoters using an in vivo reference standard. J. Biol. Eng.

[27] Rudge T. J., Brown J. R., Federici F., Dalchau N., Phillips A., Ajioka J. W., Haseloff J. (2016). Characterization
of Intrinsic Properties
of Promoters. ACS Synth. Biol.

[28] Guiziou S., Sauveplane V., Chang H. J., Clerte C., Declerck N., Jules M., Bonnet J. (2016). A part toolbox to tune genetic expression
in Bacillus subtilis. Nucleic Acids Res.

[29] Beal J., Telmer C. A., Vignoni A., Boada Y., Baldwin G. S., Hallett L., Lee T., Selvarajah V., Billerbeck S., Brown B. (2022). Multicolor
plate reader
fluorescence calibration. Synth. Biol.

[30] Torella J. P., Lienert F., Boehm C. R., Chen J. H., Way J. C., Silver P. A. (2014). Unique nucleotide
sequence-guided assembly of repetitive
DNA parts for synthetic biology applications. Nat. Protoc.

[31] Carroll P., Schreuder L. J., Muwanguzi-Karugaba J., Wiles S., Robertson B. D., Ripoll J., Ward T. H., Bancroft G. J., Schaible U. E., Parish T. (2010). Sensitive Detection of Gene Expression in Mycobacteria
under Replicating and Non-Replicating Conditions Using Optimized Far-Red
Reporters. PLoS One.

[32] Huff J., Czyz A., Landick R., Niederweis M. (2010). Taking phage
integration to the next level as a genetic tool for mycobacteria. Gene.

[33] Ehrt S., Guo X. V., Hickey C. M., Ryou M., Monteleone M., Riley L. W., Schnappinger D. (2005). Controlling
gene expression in mycobacteria
with anhydrotetracycline and Tet repressor. Nucleic Acids Res.

[34] Pedelacq J.
D., Cabantous S., Tran T., Terwilliger T. C., Waldo G. S. (2006). Engineering and
characterization of a superfolder green
fluorescent protein. Nat. Biotechnol.

[35] Halleran A.
D., Swaminathan A., Murray R. M. (2018). Single Day Construction of Multigene
Circuits with 3G Assembly. ACS Synth. Biol.

[36] Li X., Mei H., Chen F., Tang Q., Yu Z., Cao X., Andongma B. T., Chou S. H., He J. (2017). Transcriptome Landscape
of *Mycobacterium smegmatis*. Front. Microbiol.

[37] Martini M. C., Zhou Y., Sun H., Shell S. S. (2019). Defining the Transcriptional
and Post-transcriptional Landscapes of *Mycobacterium smegmatis* in Aerobic Growth and Hypoxia. Front. Microbiol.

[38] Shimada T., Makinoshima H., Ogawa Y., Miki T., Maeda M., Ishihama A. (2004). Classification
and strength measurement of stationary-phase
promoters by use of a newly developed promoter cloning vector. J. Bacteriol.

[39] Yu X., Xu J., Liu X., Chu X., Wang P., Tian J., Wu N., Fan Y. (2016). Identification
of a highly efficient stationary phase
promoter in Bacillus subtilis. Sci. Rep.

[40] Cleveland W. S., Devlin S. J. (1988). Locally Weighted
Regression - an Approach to Regression-Analysis
by Local Fitting. J. Am. Stat. Assoc.

[41] Fedorec A.
J. H., Robinson C. M., Wen K. Y., Barnes C. P. (2020). FlopR: An Open Source
Software Package for Calibration and Normalization of Plate Reader
and Flow Cytometry Data. ACS Synth. Biol.

[42] Beal J., Goni-Moreno A., Myers C., Hecht A., de Vicente M. D. C., Parco M., Schmidt M., Timmis K., Baldwin G., Friedrichs S. (2020). The long journey towards standards for engineering
biosystems: Are the Molecular Biology and the Biotech communities
ready to standardise?. EMBO Rep.

[43] Lux M. W., Strychalski E. A., Vora G. J. (2023). Advancing reproducibility can ease
the ‘hard truths’ of synthetic biology. Synth. Biol.

[44] Raj A., van Oudenaarden A. (2008). Nature, nurture, or chance: Stochastic gene expression
and its consequences. Cell.

[45] Van
Assche E., Van Puyvelde S., Vanderleyden J., Steenackers H. P. (2015). RNA-binding proteins involved in post-transcriptional
regulation in bacteria. Front. Microbiol.

[46] Liu Q., Li D., Wang N., Guo G., Shi Y., Zou Q., Zhang X. (2022). Identification and
Application of a Panel of Constitutive Promoters
for Gene Overexpression in *Staphylococcus aureus*. Front. Microbiol.

[47] Wilson E. H., Groom J. D., Sarfatis M. C., Ford S. M., Lidstrom M. E., Beck D. A. C. (2021). A Computational Framework for Identifying Promoter
Sequences in Nonmodel Organisms Using RNA-seq Data Sets. ACS Synth. Biol.

[48] Shi T., Zhang L., Liang M., Wang W., Wang K., Jiang Y., Liu J., He X., Yang Z., Chen H. (2021). Screening and engineering
of high-activity promoter
elements through transcriptomics and red fluorescent protein visualization
in *Rhodobacter sphaeroides*. Synth. Syst. Biotechnol.

[49] Luo Y., Zhang L., Barton K. W., Zhao H. (2015). Systematic Identification
of a Panel of Strong Constitutive Promoters from Streptomyces albus. ACS Synth. Biol.

[50] Born J., Pfeifer F. (2019). Improved GFP Variants
to Study Gene Expression in Haloarchaea. Front.
Microbiol.

[51] Gefen O., Fridman O., Ronin I., Balaban N. Q. (2014). Direct observation
of single stationary-phase bacteria reveals a surprisingly long period
of constant protein production activity. Proc.
Natl. Acad. Sci. U. S. A.

[52] Bush M. J. (2018). The actinobacterial
WhiB-like (Wbl) family of transcription factors. Mol. Microbiol.

[53] Lee J. Y., Park J. S., Kim H. J., Kim Y., Lee H. S. (2012). Corynebacterium
glutamicum whcB, a stationary phase-specific regulatory gene. FEMS Microbiol. Lett.

[54] Liebeke M., Brozel V. S., Hecker M., Lalk M. (2009). Chemical characterization
of soil extract as growth media for the ecophysiological study of
bacteria. Appl. Microbiol. Biotechnol.

[55] Vilain S., Luo Y., Hildreth M. B., Brozel V. S. (2006). Analysis of the life cycle of the
soil saprophyte Bacillus cereus in liquid soil extract and in soil. Appl. Environ. Microbiol.

[56] Sureka K., Ghosh B., Dasgupta A., Basu J., Kundu M., Bose I. (2008). ositive feedback and
noise activate the stringent response regulator
rel in mycobacteria. PLoS One.

[57] Keren L., Zackay O., Lotan-Pompan M., Barenholz U., Dekel E., Sasson V., Aidelberg G., Bren A., Zeevi D., Weinberger A. (2013). Promoters maintain their relative activity levels under different
growth conditions. Mol. Syst. Biol.

[58] Manganelli R., Proveddi R., Rodrigue S., Beaucher J., Gaudreau L., Smith I. (2004). σ Factors and
Global Gene Regulation in Mycobacterium tuberculosis. J. Bacteriol.

[59] Newton-Foot M., Gey van Pittius N. C. (2013). The complex architecture of mycobacterial
promoters. Tuberculosis.

[60] Zaragoza-Contreras R., Aguilar-Ayala D. A., Garcia-Morales L., Ares M. A., Helguera-Repetto A. C., Cerna-Cortes J. F., Leon-Solis L., Suarez-Sanchez F., González-Y-Merchand J. A., Rivera-Gutierrez S. (2024). Novel Populations
of *Mycobacterium smegmatis* Under Hypoxia and Starvation:
Some Insights on Cell Viability and Morphological Changes. Microorganisms.

[61] Kim N. K., Baek J. E., Lee Y. J., Oh Y., Oh J. I. (2024). Rel-dependent
decrease in the expression of ribosomal protein genes by inhibition
of the respiratory electron transport chain in *Mycobacterium
smegmatis*. Front. Microbiol.

[62] Che, A. Part:PSB1C3, 2008. https://parts.igem.org/Part:pSB1C3.

[63] Eitson J. L., Medeiros J. J., Hoover A. R., Srivastava S., Roybal K. T., Ainsa J. A., Hansen E. J., Gumbo T., van Oers N. S. C. (2012). Mycobacterial shuttle vectors designed
for high-level
protein expression in infected macrophages. Appl. Environ. Microbiol.

[64] Beggs M. L., Crawford J. T., Eisenach K. D. (1995). Isolation and sequencing of the replication
region of Mycobacterium avium plasmid pLR7. J. Bacteriol.

[65] Fernandez-Velez I., Bidegain G., Ben-Horin T. (2023). Predicting
the Growth of Vibrio parahaemolyticus
in Oysters under Varying Ambient Temperature. Microorganisms.

[66] RStudio: Integrated Development Environment for R. Posit Software; 2024. http://www.posit.co/.

[67] ggbump: Bump Chart and Sigmoid Curves; 2025. https://github.com/davidsjoberg/ggbump.

[68] ggplot2: Elegant Graphics for Data Analysis; 2016. https://ggplot2.tidyverse.org.

[69] ggpubr: ‘ggplot2’ Based Publication Ready Plots; 2023. https://rpkgs.datanovia.com/ggpubr/.

[70] Wickham H., Averick M., Bryan J., Chang W., McGowan L., François R., Grolemund G., Hayes A., Henry L., Hester J. (2019). Welcome to the Tidyverse. J.
Open Source Softw.

[71] Lassmann T., Sonnhammer E. L. (2005). Kalign–an accurate and fast multiple sequence
alignment algorithm. BMC Bioinf.

[72] Procter J. B., Carstairs G. M., Soares B., Mourao K., Ofoegbu T. C., Barton D., Lui L., Menard A., Sherstnev N., Roldan-Martinez D. (2021). Alignment of Biological Sequences with
Jalview. Methods Mol. Biol.

