# Supplementary material for: Construction of a Mycobacterium smegmatis Promoter Library for Therapeutic and Environmental Applications
Source: ACS Omega. 2025 Oct 2;10(40):46457–66. doi: 10.1021/acsomega.5c02222 (PMC12529400; doi:10.1021/acsomega.5c02222)
Supplement: Supplementary file 1 [file ao5c02222_si_001.pdf]

Supporting Information

Construction of a *Mycobacterium smegmatis* Promoter Library for Therapeutic and Environmental Applications

Lin Fang [a#], Elias H. Nafziger [b%], Min Guo [a&], Margaret S. Saha [c*]

[a] Computational & Applied Mathematics & Statistics, William & Mary, Williamsburg, VA 23185, United States
[b] Department of Biology, William & Mary, Williamsburg, VA 23185, United States
[c] Department of Applied Science, William & Mary, Williamsburg, VA 23185, United States
[*] Email: mssaha@wm.edu

[#] Systems, Synthetic, and Physical Biology, Rice University, 6100 Main St, Houston, TX 77005, United States
[%] Virology, Immunology, and Microbiology, Boston University Chobanian and Avedisian School of Medicine, 620 Albany St, Boston, MA 02218, United States
[&] Biostatistics and Health Data Science, University of Minnesota Twin Cities Graduate School, 321 Johnston Hall 101 Pleasant Street SE, Minneapolis, MN 55455, United States

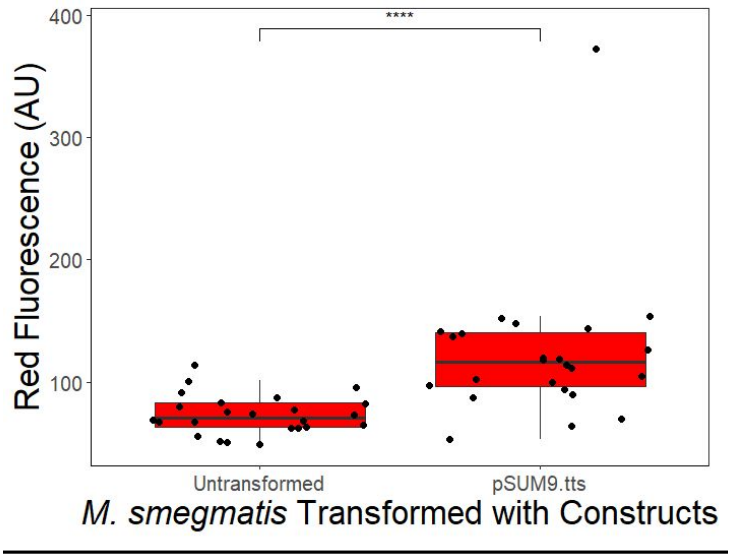

Figure S1. Background red fluorescence of *M. smegmatis* MC²155 and *M. smegmatis* MC²155 transformed with the negative control construct. *M. smegmatis* transformed with the negative control plasmid pSUM.tts exhibits red fluorescence significantly higher than untransformed *M. smegmatis* (the Wilcoxon rank-sum test, *****P* = 0.0042). n=24.

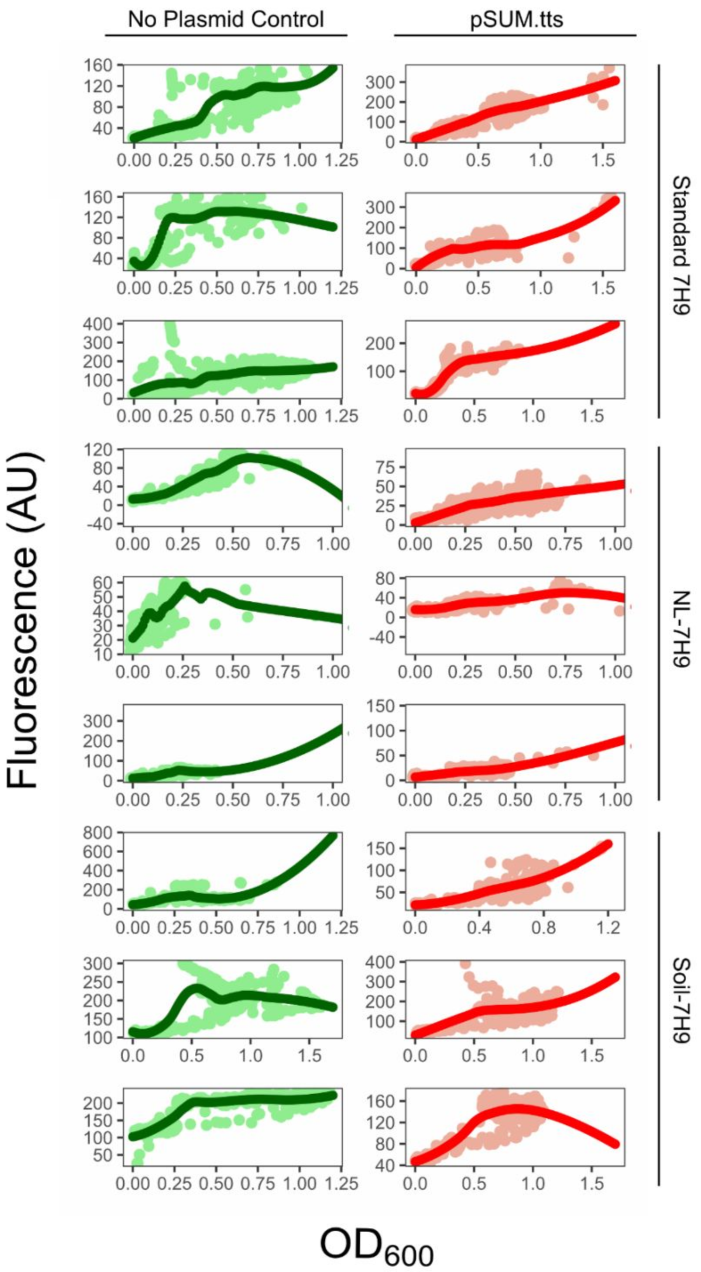

Figure S2. LOESS Models used for Background Normalization. A total of 18 LOESS models were constructed for normalizing transcription unit (TU) outputs in this study. Green fluorescence of untransformed *Mycobacterium smegmatis* was used for normalizing control TU outputs. Red fluorescence of *M. smegmatis* transformed with pSUM.tts was used for normalizing test TU outputs. For each plate run (three plates per growth condition), six biological replicates of untransformed *M. smegmatis* and *M. smegmatis* transformed with pSUM.tts were included for constructing LOESS models specific to each plate.

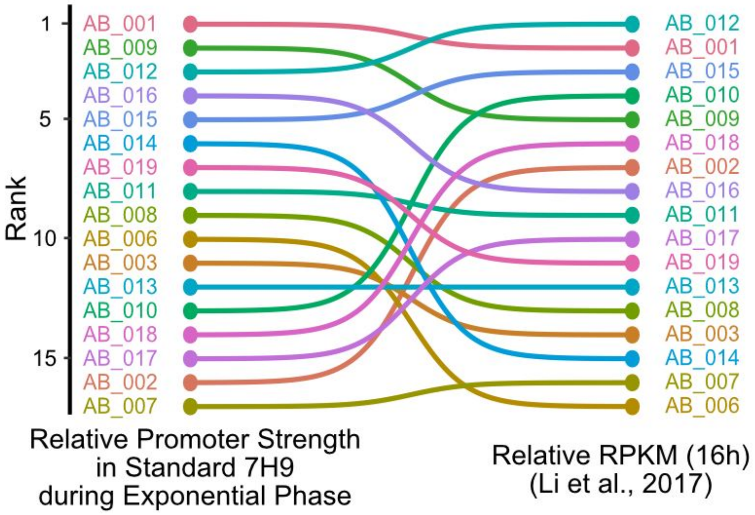

Figure S3. Change of rank orders of test promoters from the rank order determined by relative promoter strength in standard 7H9 during exponential growth to the rank order determined by relative RPKM (16h) from Li et al. (2017).[1]

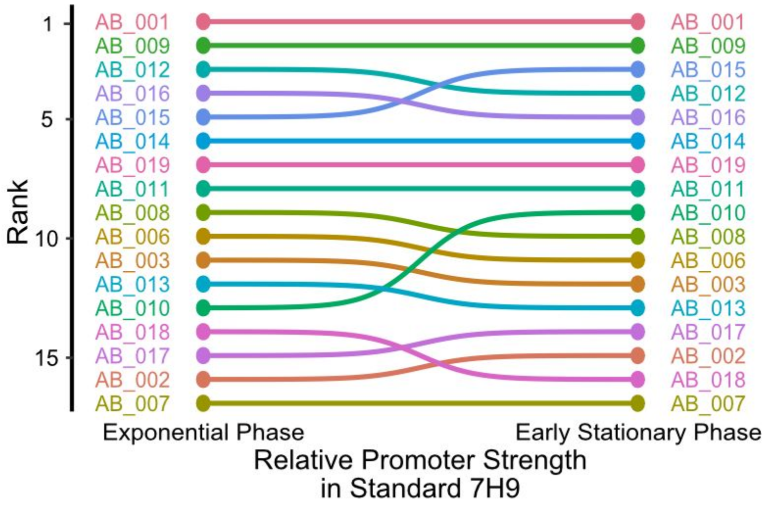

Figure S4. Change of rank orders of test promoters from the rank order determined by relative promoter strengths in standard 7H9 during the exponential growth phase to the rank order of relative promoter strengths in standard 7H9 at early stationary phase.

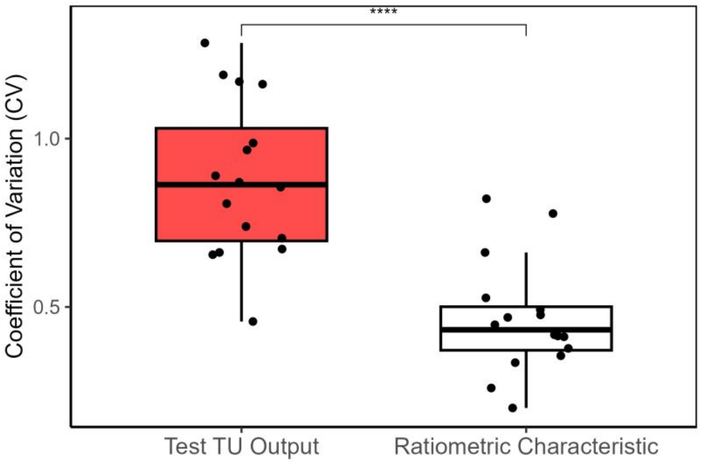

Figure S5. Comparison of the coefficient of variations (CV) of the distribution of promoter characteristic measurements using single-channel test TU outputs and dual-channel ratiometric characteristics. CV is defined as sd/mean. Each point represents the CV of the distribution of a promoter construct's characteristics measured under all culturing conditions pooled. The Kruskal-Wallis rank-sum test, ****p < 0.001. n = 6 per promoter construct.

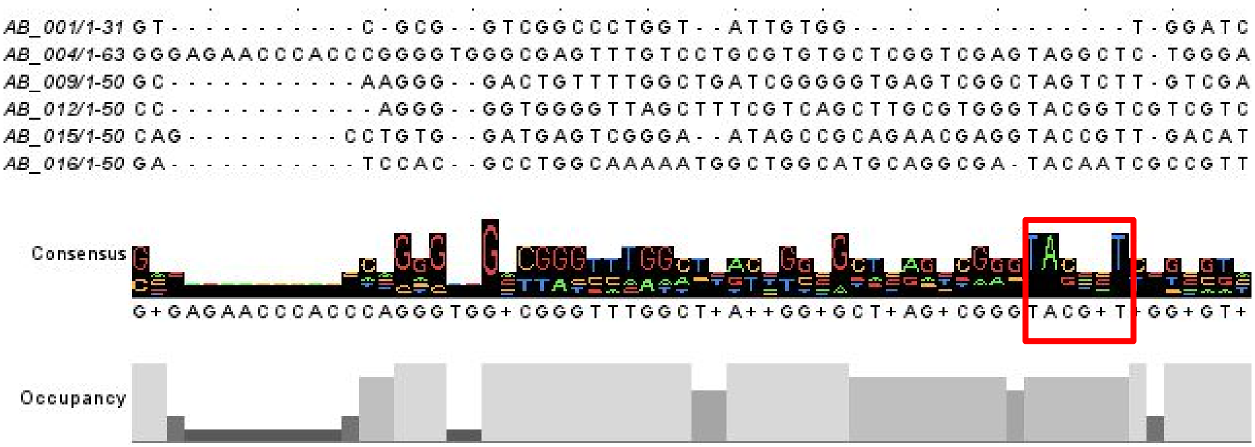

Figure S6. Multiple sequence alignment of strong promoters. Putative -10 region is boxed in red.

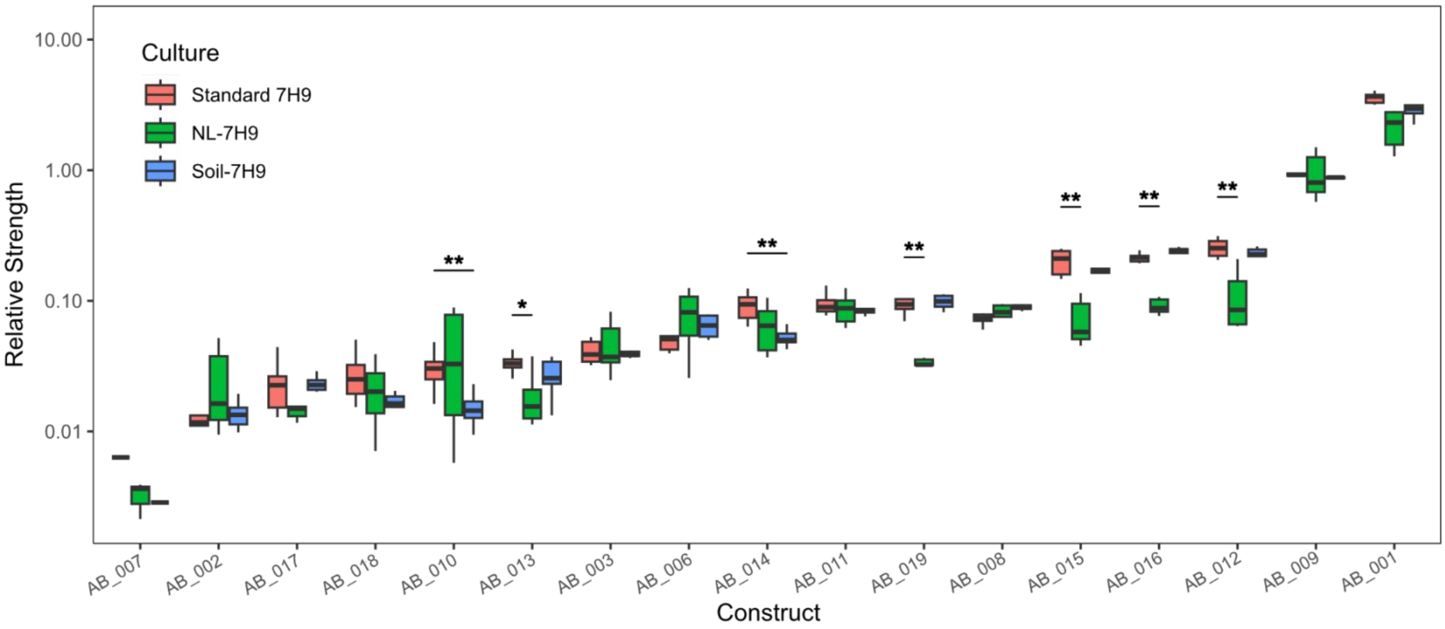

Figure S7. Promoter Relative Strengths Across Culturing Conditions. Relative Strengths were calculated by normalizing each promoter's ratiometric characteristics to the mean of the AB_004's ratiometric characteristics measured under the appropriate culturing condition. Thirteen negative data points of AB_007 and one negative data point of AB_002 are omitted after the log transformation of relative strengths. n=6. Wilcoxon rank-sum test, ** $p < 0.0087$ for AB_010 (Standard vs. Soil), ** $p < 0.0043$ for AB_012 (Standard vs. NL), * $p < 0.041$ for AB_013 (Standard vs. NL),, ** $p < 0.0043$ for AB_014 (Standard vs. Soil), ** $p < 0.0022$ for AB_015 (Standard vs. NL), ** $p < 0.0022$ for AB_016 (Standard vs. NL), ** $p < 0.0022$ for AB_019 (Standard vs. NL).

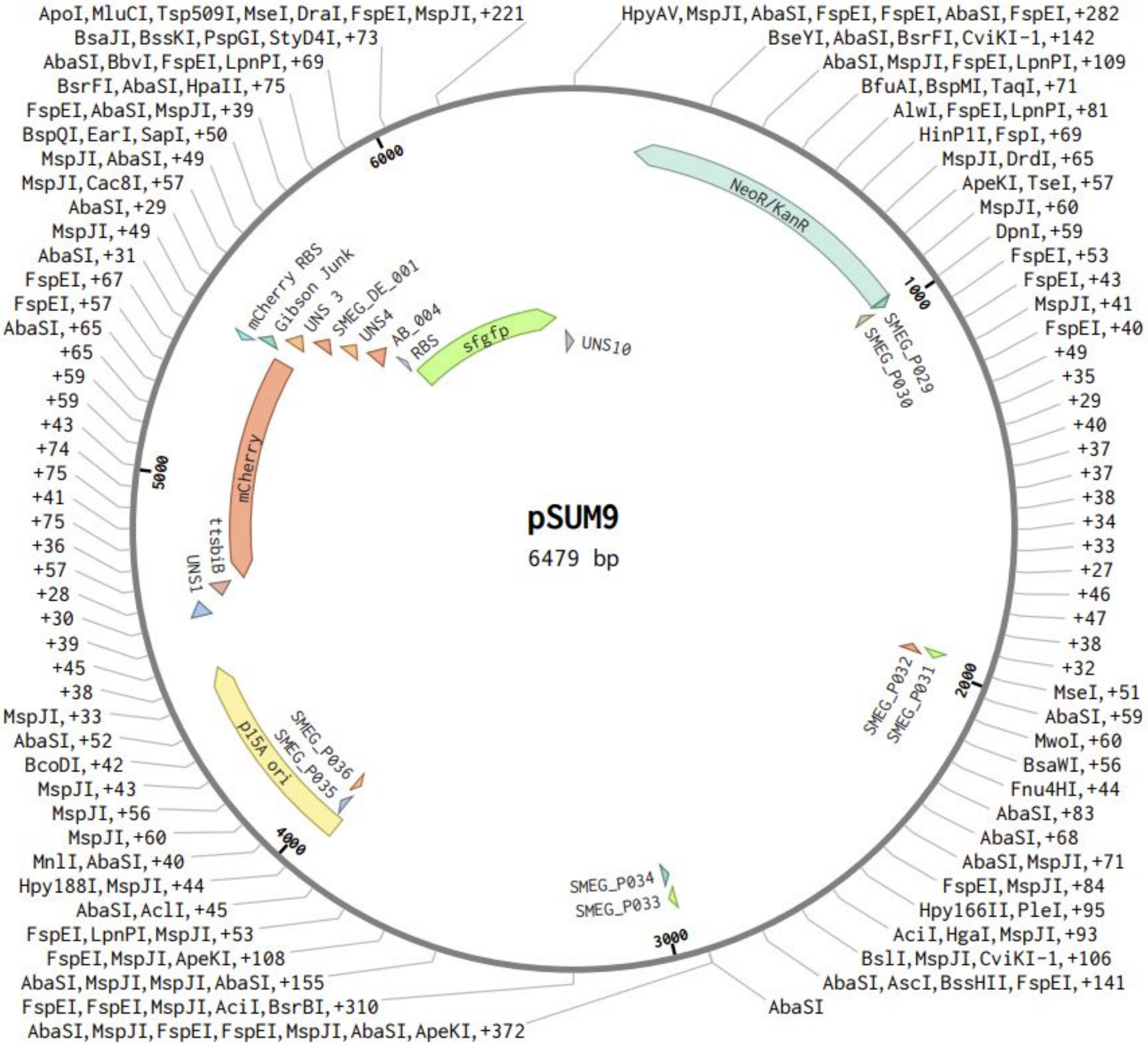

Figure S8. pSUM9 plasmid map from Benchling [Biology Software] (2024). pSUM9 contains BsaI cut sites suitable for the scarless insertion of test promoters.

```
cgaagaactccagcatgagatccccgcgctggaggatcatccagccggcgtcccggaaaacgattccgaagcccaacctttcat
agaaggcggcggtggaatcgaaatctcgtgatggcaggttgggcgtcgcttggtcggtcatttcgaaccccagagtcccgctcag
aagaactcgtcaagaaggcgatagaaggcgatgcgctgcgaatcgggagcggcgataccgtaaagcacgaggaagcggtca
gcccattcgccgccaagctcttcagcaatatcacgggtagccaacgctatgtcctgatagcggtccgccacacccagccggccac
agtcgatgaatccagaaaagcggccattttccaccatgatattcggcaagcaggcatcgccatgggtcacgacgagatcctcgcc
gtcgggcatccgcgccttgagcctggcgaacagttcggctggcgcgagcccctgatgctcttcgtccagatcatcctgatcgacaa
gaccggcttccatccgagtacgtgctcgctcgatgcgatgtttcgcttggtggtcgaatgggcaggtagccggatcaagcgtatgca
gccgccgcattgcatcagccatgatggatactttctcggcaggagcaaggtgagatgacaggagatcctgccccggcacttcgcc
caatagcagccagtcccttcccgcttcagtgacaacgtcgagcacagctgcgcaaggaacgcccgtcgtggccagccacgata
gccgcgctgcctcgtcttggagttcattcagggcaccggacaggtcggtcttgacaaaaagaaccgggcgcccctgcgctgacag
ccggaacacggcggcatcagagcagccgattgtctgttgtgcccagtcatagccgaatagcctctccacccaagcggccggaga
acctgcgtgcaatccatcttgttcaatcatgcgaaacgatcctcatcctgtctcttgatcagatcttgatcccctgcgccatcagatcctt
ggcggcaagaaagccatccagtttactttgcagggcttcccaaccttaccagagggcgccccagctggcaattccggttcgcttgct
gtccataaaaccgcccagtctagctatcgccatgtaagcccactgcaagctacctgctttctctttgcgcttgcgttttcccttgtccagat
```

agcccagtagctgacattcatccggggtcagcaccgtttctgcggactggctttctacgtgttccgcttcctttagcagcccttgcgccct
gagtgcttgcggcagcgtgaagctgtgcctagaaataacgaggacagtcgcacgacgaagttcttctggatcgcgcccgtgctgg
aagcactcaacctcgaagcgtgtggttgcggagccatctagcaaccacacgaaacatgcgcaacgaaccgcgcaacgaaca
acgcctagaactggccctagatgagctgactcgtatcgttggtaaacctagtttgaccagcatgttttaactacgttcggtgagctgtc
aacggggcctgtaacggcacaacgaaccgtgcaacgagagtggccacggatgccaccacaggcactacaacggagttcgcc
acgtacatcaccacaaccaccgattctggcggtgagctccccgatattcagcggaaatggcttggtatcgaccaagattcgtagaa
ccccgtctcgtctggctggtattcaaaacgggcgcaacgaaacacgcaacgagacaggcatggcccaaaccagaaaactagc
gtctaccaggacttttacctgtccgacccgttgcaacggaaccccccacggaacccccgcgacacccgctccccaattgcgttaga
acagcggtggattgtcggcttcgttgtgggccttttgagccgcttcctgttctgccgcacgctctttcctcgcccgatagccgagtcgctt
aacggtgtccagatgcagcccgaaatgtttggccgtttgcggccaagagtggccctcgtcgtcgtgataggcgcggatgcgttcgc
ggcgtgcagcctgctcggcgagccactcgctgcgttcctgcgccacgagccggacgacgtggcgttcggatagtccggtgattcg
agcgccttcggcggcggtcacgcgccgctttttgcggacagtcggctgccggttgtagccgtcgctgtagccgtcgctcatagcaat
gcctccatggctgacgcggactttgcgcgccgcgcaactgtgctcgccgccgtgcgcgctgctgcgcccttccgcgagatggccg
actggcgcgcactgagtgtggcctcgtagaccacgatcccgtccgcccaaatgcgcgacttggttgtgatccaacgccaaatgctg
ttggcgatggcgcggacctcgctgtccggtagcggtccgggacacacgtcgttgcacgggaattcggcgtttcgcgcgtggcactc
ggcatagatcgcgcggccgagtccgtccacgttccgggtcggcaggtagatccgcatgagggcgggacgataggcccacaac
ctgacggaatcgaacagtgcgcaattccgccctagcggcgtcggagccgctttgtacgtggtctgctgacgccagcgcggcggtg
gcatgttcgcgccgagctcggcctcgatgtggctgagtgtgtagagatctgagtggagccattccgtttcccaggcgatgtggccgg
ggtttttggtcatgaggcctgagtaactgcggtcgccgtcgacggcgcgccgaaggccttcggcgcacgccgccatgtatgcgag
cggcttacgccgcgcgtattcggtgcgtggaacaggggcgttgagtgcccacactgcgtgtgcgtggccgttggcgcgattgccca
cgatcgcgttgggcagcggatgggacccccgggcgctgagcgctcggagcgctgcgtctggatggtctacgtccacgaccagca
ggtttgccagcgctgttgggttcgcctcgatgtaccggcggcctagggccgacgcgcggctttggcggtagatcccctcgagcaga
tcgtcgcttgccagcggccagtacggcagccagagctgctcaaattcgtcggcgacgtggctcacgcttggtagtagaccacgatt
aatcaccggtgtatggtccgacacgagctccaagtcagatatttcgctgaggggccaccccacaactgcacactcccccgctctcc
cgtcgagccctggtggtggaacaccagcgacagccgagcacccccaaccacctgtaccaaccaggaggaacacatgcgtcgt
ttcgaggacgtttccgggccgctgagagccgctgtggcggccgtacacgccgccttagacccgttagacccccctgccgcctgaatg
cgcgggtacgagccacacagcgcccgaacttacggagctggtgggctcacctggctttatggcgtacgaatcggctgtgtgcgac
ctgttgggcgaggtgaggtacgcgctactcacgctggcaagggcgacagagccgccccaccgagcccgcacggccgcgcgc
ggtgtcaacaaccgggtgagtcgtgcacaccagcaggtgttcgaggcttggctcgaagtgcaggacatcgtggcgaacgccgcc
cgatgagccgcgccttacgctggctgccagccgttcgcgggctggttggtgcagcgcgtcgagcggttagaggccctgcggtgttc
caccaccgcagggcctcgccctttttaaggctgaatttgcttgtctccgaatccaactggcttgtccaagggtgtatctacgcttaatcc
aaagttcaaacgaggggattacacatgaccaacttcgataacgttctcggctcgatattttatctgattaataagatgatcttcttgaga
tcgttttggtctgcgcgtaatctcttgctctgaaaacgaaaaaaccgccttgcagggcggtttttcgaaggttctctgagctaccaactct
ttgaaccgaggtaactggcttggaggagcgcagtcaccaaaacttgtcctttcagtttagccttaaccggcgcatgacttcaagacta
actcctctaaatcaattaccagtggctgctgccagtggtgcttttgcatgtctttccgggttggactcaagacgatagttaccggataag
gcgcagcggtcggactgaacggggggttcgtgcatacagtccagcttggagcgaactgcctacccggaactgagtgtcaggcgt
ggaatgagacaaacgcggccataacagcggaatgacaccggtaaaccgaaaggcaggaacaggagagcgcacgaggga
gccgccagggggaaacgcctggtatctttatagtcctgtcgggtttcgccaccactgatttgagcgtcagatttcgtgatgcttgtcagg
ggggcggagcctatggaaaaacggctttgccgcggccctctcacttccctgttaagtatcttcctggcatcttccaggaaatctccgc
cccgttcgtaagccatttccgctcgccgcagtcgaacgaccgagcgtagcgagtcagtgagcgaggaagcggaatacattactc
gcatccattctcaggctgtctcgtctcgtctcaagcaaaaaaaaaagcgccgcaactgcggcgctttttttttaccttcacttgtacagc
tcgtccatgccgccggtgctgtggcggccctcggcgcgctcgtactgctcgacgatggtgtagtcctcgttgtggctggtgatgtccag
cttgatgttcacgttgtaggcgcccggcagctggaccggcttcttggccttgtaggtggtcttgacctcggcgtcgtagtggccgccgtc
cttcagcttcagccgctgcttgatctcgcccttcagggcgccgtcctccgggtacatgcgctccgacgaggcctcccagcccatggtc
ttcttctgcatgaccgggccgtccgacgggaagttggtgccccgcagcttgaccttgtagatgaactcgccgtcctgcagcgacgag
tcctgggtgacggtgacgacgccgccgtcctcgaagttcatgacgcgctcccacttgaagccctccgggaacgacagcttcaggt
agtccgggatgtcggccgggtgcttgacgtaggccttgctgccgtacatgaactgcggcgacaggatgtcccaggcgaacggca
gcgggccgcccttggtgaccttcagcttggcggtctgggtgccctcgtacgggcggccctcgccctcgccctcgatctcgaactcgt
ggccgttgaccgagccctccatgtggaccttgaagcgcatgaactccttgatgatcgccattgttgtcctcctcgccctagtaagaga
cc**ataatgataaatcgagatctatgag**ggtctcactccgcactgaaggtcctcaatcgcactggaaacatcaaggtcgaggtaa

aaaaaaaagcgccgcagttgcggcgcttttttttttgcttctgacctcctgccagcaatagtaagacaacacgcaaagtcggaggg
gagaacccacccggggtgggcgagtttgtcctgcgtgtgctcggtcgagtaggctctgggatactagggcgaggaggacaacaa
tgcgtaaaggcgaagagctgttcactggtgtcgtccctattctggtggaactggatggtgatgtcaacggtcataagttttccgtgcgtg
gcgagggtgaaggtgacgcaactaatggtaaactgacgctgaagttcatctgtactactggtaaactgccggtaccttggccgact
ctggtaacgacgctgacttatggtgttcagtgctttgctcgttatccggaccatatgaagcagcatgacttcttcaagtccgccatgccg
gaaggctatgtgcaggaacgcacgatttcctttaaggatgacggcacgtacaaaacgcgtgcggaagtgaaatttgaaggcgat
accctggtaaaccgcattgagctgaaaggcattgactttaaagaagatggcaatatcctgggccataagctggaatacaattttaac
agccacaatgtttacatcaccgccgataaacaaaaaaatggcattaaagcgaattttaaaattcgccacaacgtggaggatggca
gcgtgcagctggctgatcactaccagcaaaacactccaatcggtgatggtcctgttctgctgccagacaatcactatctgagcacgc
aaagcgttctgtctaaagatccgaacgagaaacgcgatcatatggttctgctggagttcgtaaccgcagcgggcatcacgcatggt
atggatgaactgtacaaatgatgaaggtaaaaaaaaaagcgccgcagttgcggcgcttttttttttgcttccaggatacatagattac
cacaactccgagcccttccacc

Sequence S1. pSUM9 whole plasmid sequence. The gibson adaptor is highlighted in red. BsaI cut sites are highlighted in green.

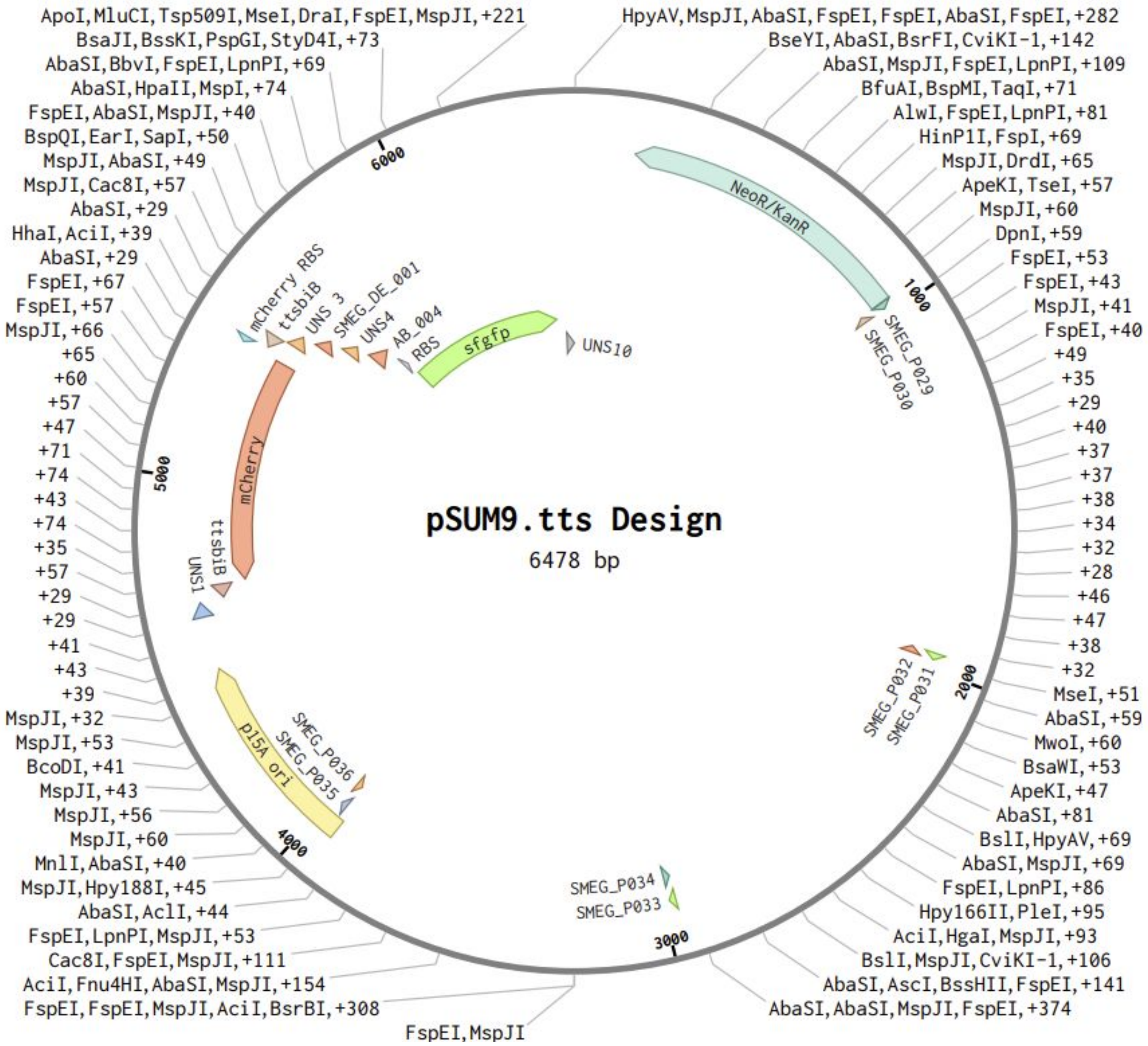

Figure S9. pSUM9.tts plasmid map. pSUM9.tts contains a bidirectional terminator in place of a test promoter, serving as the negative control for normalizing test TU outputs.

gaagcgcatgaactccttgatgatcgccattgttgtcctcctcgccctagta**aaaaaaaaaagcgccgcaactgcggcgcttttttt tttt**ctccgcactgaaggtcctcaatcgcactggaaacatcaaggtcg

Sequence S2. pSUM9.tts sequence around the inserted terminator region. The terminator sequence in place of a promoter is highlighted in red.

gaagcgcatgaactccttgatgatcgccattgttgtcctcctcgccctagtagatccaccacaataccagggccgaccgcgacc tccgcactgaaggtcctcaatcgcactggaaacatcaaggtcg

Sequence S3. pSUM9.01 sequence around the test promoter region.

gaagcgcatgaactccttgatgatcgccattgttgtcctcctcgccctagta**aaacagaacggtagggggcggacgcggctga**
tcgtggcaggctccgcactgaaggtcctcaatcgcactggaaacatcaaggtcg

Sequence S4. pSUM9.02 sequence around the test promoter region.

gaagcgcatgaactccttgatgatcgccattgttgtcctcctcgccctagta**gcccgcagcataactgaaacacgttccagtttcg**
atggtgagcggcgggcctccgcactgaaggtcctcaatcgcactggaaacatcaaggtcg

Sequence S5. pSUM9.03 sequence around the test promoter region.

ggaccttgaagcgcatgaactccttgatgatcgccattgttgtcctcctcgccctagta**tcccagagcctactcgaccgagcaca**
cgcaggacaaactcgcccaccccgggtgggttctcccctccgcactgaaggtcctcaatcgcactggaaacatcaaggtcg

Sequence S6. pSUM9.04 sequence around the test promoter region.

gaagcgcatgaactccttgatgatcgccattgttgtcctcctcgccctagta**gatcctcggtcgacgtgtgcgcgtagccgctgc**
gccagaacacccgcatcgcggtgtccagtgcgacgtccggatcgaactccttgtgacgggccatgtgccaattctagat
cgctccgcactgaaggtcctcaatcgcactggaaacatcaaggtcga

Sequence S7. pSUM9.06 sequence around the test promoter region.

ggaccttgaagcgcatgaactccttgatgatcgccattgttgtcctcctcgccctagta**agttccacggtaaatacgcaggtcacg**
tgcgtgcagtgcatgtactccgcactgaaggtcctcaatcgcactggaaacatcaaggtcg

Sequence S8. pSUM9.07 sequence around the test promoter region.

gaagcgcatgaactccttgatgatcgccattgttgtcctcctcgccctagta**gtcctgtcgataaccgctttacgcgctcaccaaa**
ccggtcagcctatggcctccgcactgaaggtcctcaatcgcactggaaacatcaaggtcg

Sequence S9. pSUM9.08 sequence around the test promoter region.

gaagcgcatgaactccttgatgatcgccattgttgtcctcctcgccctagta**tcgacaagactagccgactcacccccgatcagc**
caaaacagtccccttgcctccgcactgaaggtcctcaatcgcactggaaacatcaaggtcg

Sequence S10. pSUM9.09 sequence around the test promoter region.

gaagcgcatgaactccttgatgatcgccattgttgtcctcctcgccctagta**actacagtgatacccacctgcgccctcgattaag**
cgtgtgatatgcgtctctccgcactgaaggtcctcaatcgcactggaaacatcaaggtcg

Sequence S11. pSUM9.10 sequence around the test promoter region.

gaagcgcatgaactccttgatgatcgccattgttgtcctcctcgccctagta**gtccccaaattacgtgcgcgagaggaagcgcg**
ccagaaccaccgcgcgtgctccgcactgaaggtcctcaatcgcactggaaacatcaaggtcg

Sequence S12. pSUM9.11 sequence around the test promoter region.

gaagcgcatgaactccttgatgatcgccattgttgtcctcctcgccctagta**gacgacgaccgtacccacgcaagctgacgaaa**
gctaaccccacccccctggctccgcactgaaggtcctcaatcgcactggaaacatcaaggtcg

Sequence S13. pSUM9.12 sequence around the test promoter region.

ggaccttgaagcgcatgaactccttgatgatcgccattgttgtcctcctcgccctagta**gatcggcagaatatccccagcgacgct**
cggacaaacgcgttcgcgtatcactccgcactgaaggtcctcaatcgcactggaaacatcaaggtcg

Sequence S14. pSUM9.13 sequence around the test promoter region.

gaagcgcatgaactccttgatgatcgccattgttgtcctcctcgccctagta**accaccattatccccggtcgcggtcaagacgtg**
ggtgaacaccgcgagtgctccgcactgaaggtcctcaatcgcactggaaacatcaaggtcg

Sequence S15. pSUM9.14 sequence around the test promoter region.

gaagcgcatgaactccttgatgatcgccattgttgtcctcctcgccctagta**atgtcaacggtacctcgttctgcggctattcccga**
ctcatccacaggctgctccgcactgaaggtcctcaatcgcactggaaacatcaaggtcg

Sequence S16. pSUM9.15 sequence around the test promoter region.

gaagcgcatgaactccttgatgatcgccattgttgtcctcctcgccctagta**aacggcgattgtatcgcctgcatgccagccatttt**
tgccaggcgtggatcctccgcactgaaggtcctcaatcgcactggaaacatcaaggtcg

Sequence S17. pSUM9.16 sequence around the test promoter region.

gaagcgcatgaactccttgatgatcgccattgttgtcctcctcgccctagta**acccgacgttatcgcgtcagcgaacaaacgca**
ggtgaacctcactggatcctccgcactgaaggtcctcaatcgcactggaaacatcaaggtcg

Sequence S18. pSUM9.17 sequence around the test promoter region.

ggaccttgaagcgcatgaactccttgatgatcgccattgttgtcctcctcgccctagta**gactctgagcgtatgggtcactattaag**
gaatctttgagtcacgattcggctccgcactgaaggtcctcaatcgcactggaaacatcaaggtcg

Sequence S19. pSUM9.18 sequence around the test promoter region.

gaagcgcatgaactccttgatgatcgccattgttgtcctcctcgccctagta**gcggttcatgttgccatgcgggcacggggtaaa**
cgatccgggccagtgaactccgcactgaaggtcctcaatcgcactggaaacatcaaggtcg

Sequence S20. pSUM9.19 sequence around the test promoter region.

References

(1) Li, X.; Mei, H.; Chen, F.; Tang, Q.; Yu, Z.; Cao, X.; Andongma, B. T.; Chou, S. H.; He, J. Transcriptome Landscape of Mycobacterium smegmatis. *Front Microbiol* **2017**, *8*, 2505. DOI: 10.3389/fmicb.2017.02505

(2) Kenney, T. J.; Churchward, G. Genetic analysis of the *Mycobacterium smegmatis* rpsL promoter. *J Bacteriol* **1996**, *178* (12), 3564-3571. DOI: 10.1128/jb.178.12.3564-3571

(3) Roy, S.; Anand, D.; Vijay, S.; Gupta, P.; Ajitkumar, P. The ftsZ Gene of *Mycobacterium smegmatis* is expressed Through Multiple Transcripts. *Open Microbiol J* **2011**, *5*, 43-53. DOI: 10.2174/1874285801105010043

(4) Uhia, I.; Galan, B.; Medrano, F. J.; Garcia, J. L. Characterization of the KstR-dependent promoter of the gene for the first step of the cholesterol degradative pathway in *Mycobacterium smegmatis*. *Microbiology (Reading)* **2011**, *157* (Pt 9), 2670-2680. DOI: 10.1099/mic.0.049213-0

(5) Kaps, I.; Ehrt, S.; Seeber, S.; Schnappinger, D.; Martin, C.; Riley, L. W.; Niederweis, M. Energy transfer between fluorescent proteins using a co-expression system in *Mycobacterium smegmatis. Gene* **2001**, *278* (1-2), 115-124. DOI: 10.1016/s0378-1119(01)00712-0

(6) Ehrt, S.; Guo, X. V.; Hickey, C. M.; Ryou, M.; Monteleone, M.; Riley, L. W.; Schnappinger, D. Controlling gene expression in mycobacteria with anhydrotetracycline and Tet repressor. *Nucleic Acids Res* **2005**, *33* (2), e21. DOI: 10.1093/nar/gni013

(7) Martini, M. C.; Zhou, Y.; Sun, H.; Shell, S. S. Defining the Transcriptional and Post-transcriptional Landscapes of Mycobacterium smegmatis in Aerobic Growth and Hypoxia. *Front Microbiol* **2019**, *10*, 591. DOI: 10.3389/fmicb.2019.00591

(8) Carroll, P.; Schreuder, L. J.; Muwanguzi-Karugaba, J.; Wiles, S.; Robertson, B. D.; Ripoll, J.; Ward, T. H.; Bancroft, G. J.; Schaible, U. E.; Parish, T. Sensitive detection of gene expression in mycobacteria under replicating and non-replicating conditions using optimized far-red reporters. *PLoS One* **2010**, *5* (3), e9823. DOI: 10.1371/journal.pone.0009823

(9) Pedelacq, J. D.; Cabantous, S.; Tran, T.; Terwilliger, T. C.; Waldo, G. S. Engineering and characterization of a superfolder green fluorescent protein. *Nat Biotechnol* **2006**, *24* (1), 79-88. DOI: 10.1038/nbt1172

(10) Huff, J.; Czyz, A.; Landick, R.; Niederweis, M. Taking phage integration to the next level as a genetic tool for mycobacteria. *Gene* **2010**, *468* (1-2), 8-19. DOI: 10.1016/j.gene.2010.07.012

(11) Torella, J. P.; Lienert, F.; Boehm, C. R.; Chen, J. H.; Way, J. C.; Silver, P. A. Unique nucleotide sequence-guided assembly of repetitive DNA parts for synthetic biology applications. *Nat Protoc* **2014**, *9* (9), 2075-2089. DOI: 10.1038/nprot.2014.145
